# Substrate regulation leads to differential responses of microbial ammonia-oxidizing communities to ocean warming

**DOI:** 10.1038/s41467-020-17366-3

**Published:** 2020-07-14

**Authors:** Zhen-Zhen Zheng, Li-Wei Zheng, Min Nina Xu, Ehui Tan, David A. Hutchins, Wenchao Deng, Yao Zhang, Dalin Shi, Minhan Dai, Shuh-Ji Kao

**Affiliations:** 1 State Key Laboratory of Marine Environmental Science, College of the Environment and Ecology, Xiamen University, Xiamen, Fujian P. R. China; 2 State Key Laboratory of Marine Environmental Science, College of Ocean and Earth Sciences, Xiamen University, Xiamen, Fujian P. R. China; 3 Marine and Environmental Biology, University of Southern California, Los Angeles, CA 90089 USA

**Keywords:** Element cycles, Biogeochemistry, Marine chemistry

## Abstract

In the context of continuously increasing anthropogenic nitrogen inputs, knowledge of how ammonia oxidation (AO) in the ocean responds to warming is crucial to predicting future changes in marine nitrogen biogeochemistry. Here, we show divergent thermal response patterns for marine AO across a wide onshore/offshore trophic gradient. We find ammonia oxidizer community and ambient substrate co-regulate optimum temperatures (T_opt_), generating distinct thermal response patterns with T_opt_ varying from ≤14 °C to ≥34 °C. Substrate addition elevates T_opt_ when ambient substrate is unsaturated. The thermal sensitivity of kinetic parameters allows us to predict responses of both AO rate and T_opt_ at varying substrate and temperature below the critical temperature. A warming ocean promotes nearshore AO, while suppressing offshore AO. Our findings reconcile field inconsistencies of temperature effects on AO, suggesting that predictive biogeochemical models need to include such differential warming mechanisms on this key nitrogen cycle process.

## Abstract (web summary)

Microbial ammonia oxidation is important in marine nutrient cycling and greenhouse gas dynamics, but the responses to ocean warming are unclear. Here coast to open ocean incubations show that projected year 2100 temperatures might be too hot for these microbes in oligotrophic regions to handle, but may facilitate oxidation rates in coastal waters.

## Introduction

Ammonia oxidation (AO), the first step in nitrification, connects the most reduced and oxidized inorganic nitrogen species in the ocean. It therefore replenishes the marine pools of nitrite and provides oxidized substrates for denitrification and annamox, two primary nitrogen loss terms in the ocean^1,2^. Thus, AO plays a crucial role in the marine nitrogen cycle. In addition, AO interacts with the ocean carbon cycle from various perspectives, and is therefore involved in multiple climate feedback processes. For example, chemoautotrophic ammonia-oxidizing organisms fix inorganic carbon^3^, while the end product of nitrification provides approximately half of the nitrate consumed by growing phytoplankton on a global scale^4^. On the other hand, AO generates nitrous oxide (N_2_O) as a by-product^5^, a potent greenhouse gas with a ~300-fold higher greenhouse gas potential per molecule than carbon dioxide. Thus, AO helps to make the ocean a net source of N_2_O to the atmosphere. In view of global change and the rapid increases in the influx of anthropogenic nitrogen into the marine environment^6,7^, factors like acidification^8,9^, stratification, deoxygenation, and especially warming that may affect the ammonia oxidation rate (AOR) and AO microbial community structure, need to be addressed^10,11^. Only then can these environmental change factors be properly incorporated into nitrogen-driven biogeochemical models to make accurate climate predictions.

Temperature is recognized as a primary global driver to tune biological metabolic rates^12^. However, knowledge of how marine AO responds to warming remains underexplored, especially for areas having high AOR, such as estuaries, coastal zones^13,14^, and the base of the euphotic zone in the open ocean^15–17^. These environments also appear to be the frontline of both anthropogenic nitrogen disturbances and ocean warming. Pure cultures of three strains of ammonia-oxidizing archaea (AOA) isolated from marine habitats have shown that AOA growth rates are positively correlated with temperature until their optimum temperatures (*T*_opt_) are reached^18^. Paradoxically, the limited number of field studies in marine environments^19–22^ produced inconsistent results, i.e. positive and insensitive responses to temperature increase. Due to insufficient field information, how ammonia oxidizers may respond to thermal stress in the vast ocean remains enigmatic, as do possible synergistic effects with continuously increasing anthropogenic nitrogen inputs into the ocean.

**Fig. 1:**
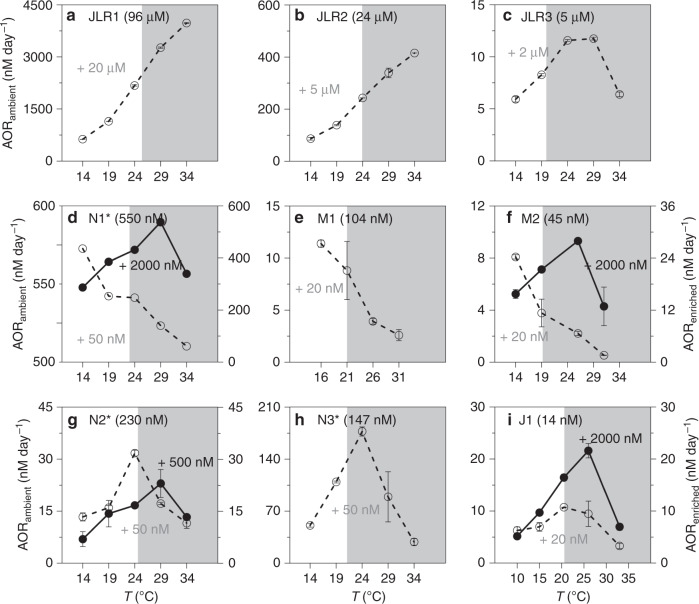
Thermal responses of ammonia oxidation rates. In situ substrate concentrations are given in parentheses. Ammonia oxidation rates near the ambient substrate level (AOR_ambient_) are shown as open circles with dashed lines and at the enriched substrate level (AOR_enriched_) as solid circles with solid lines. The tracer concentrations of ^15^NH_4_^+^ added for the ambient and enriched incubations are shown in gray and black numbers, respectively. Gray area denotes temperatures higher than the in situ temperature. The station IDs with asterisk (*) refer to the stations at which the ammonium concentrations were measured in the shore-based laboratory several days after collection. The rate data are mean values, instead of standard deviation the given bars indicate the variation range of two independent experiments, except N1 station (*n* = 1).

To better predict the future of marine nitrogen biogeochemistry, we performed manipulation experiments to characterize the temperature responses of the marine AO microbial community relative to substrate changes across a broad environmental gradient. Using isotope labeling techniques and a series of temperature/substrate manipulation incubations, we revealed distinct temperature response patterns of marine AO communities along a substrate gradient from coastal eutrophic waters to offshore oligotrophic regions (Supplementary Fig. 1; Fig. 1; Supplementary Table 1). Our experimental results shed light on the substrate-regulated thermal kinetics and parameterization of nitrification that are critically needed for marine biogeochemical models of the rapidly changing marine nitrogen cycle.

## Results and discussion

### Distinctive temperature responses along a substrate gradient

Within the temperature range of ~14 to ~34 °C in our incubations, the observed AORs at the ambient substrate level (AOR_ambient_, see Methods) varied over 3 orders of magnitude, from 0.5 to ~4000 nM d^−1^, across a wide spectrum of ambient ammonium levels ranging from 14 nM to 96 μM (Fig. 1). Three different types of temperature response of AOR_ambient_ patterns at estuarine, shelf, and sea basin stations were observed: (I) a positive response with a *T*_opt_ of ≥34 °C (Fig. 1a, b); (II) a negative response, which has never been reported before, with a *T*_opt_ of ≤14 °C (Fig. 1d–f); and (III) a dome-shaped response with a *T*_opt_ of 20–29 °C (Fig. 1c, g–i).

The Type I pattern was observed at two of the three estuarine stations (JLR1 and JLR2, Fig. 1a, b) where ammonium concentrations were high (≥24 μM), and the AOR increased linearly as the temperature increased from 14 to 34 °C. In these cases, the *T*_opt_ was equal to or higher than the maximum experimental temperature of 34 °C (Fig. 1a, b). The Type II pattern was observed at the shelf stations (N1, M1, and M2), where NH_4_^+^ concentrations ranged from 45 to 550 nM (Fig. 1d–f). In contrast to the Type I pattern, the *T*_opt_ of the Type II pattern was equal to or lower than the minimum experimental temperature of 14 °C, showing a continuously decreasing AOR as temperature increased. The Type III pattern was observed at station JLR3 (outer estuary), N2 (shelf), N3 and J1 (basin), for which the *T*_opt_ of the AOR varied from 20 to 29 °C, with rates decreasing toward both higher and lower temperatures (Fig. 1c, g–i). The NH_4_^+^ concentrations of the Type III stations ranged from 14 to 5000 nM. Nevertheless, the highest *T*_opt_ values were observed at coastal sites with the highest ambient ammonium concentrations (Fig. 1).

### Substrate regulates AOR and its thermal optimum temperature

For those stations with low ammonium concentrations, the AOR at in situ temperature increased when the substrate was enriched (AOR_enriched_, additions of 2000 nM ^15^NH_4_^+^) (Fig. 1f, i). Meanwhile, the *T*_opt_ of the AOR shifted significantly toward higher values (*t* test, *p* < 0.05; Fig. 1d, f, g, i). Although the resolution of the temperature interval set in our incubation experiments was not high enough to identify a precise *T*_opt_ for the AO community, the positive *T*_opt_ shift induced by ammonium enrichment was evident.

**Fig. 2:**
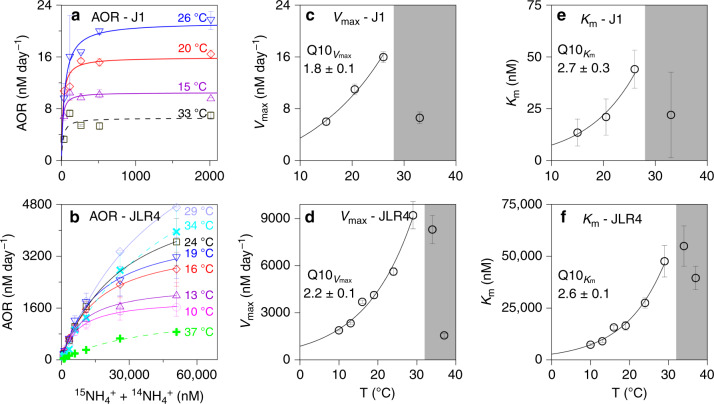
Thermal kinetics plots for ammonia oxidation rates. **a**, **b** Michaelis–Menten kinetics of ammonia oxidation rates in response to changes in temperature. **c**–**f** Arrhenius model fitting curves for the experimental *V*_max_ (J1: *Y* = (4.1 ± 0.7) × (1.8 ± 0.1)^T/10^, *R*^2^ = 0.99; JLR4: *Y* = (869.9 ± 118.2) × (2.2 ± 0.1)^T/10^, *R*^2^ = 0.98) and *K*_m_ (J1: *Y* = (2.6 ± 1.1) × (2.7 ± 0.3)^T/10^, *R*^2^ = 0.98; JLR4: *Y* = (2765.7 ± 404.6) × (2.6 ± 0.1)^T/10^, *R*^2^ = 0.99) of the ammonia oxidation rate as a function of temperature. Color curves in (**a**, **b**) represent the regression curves of the Michaelis–Menten kinetics at various temperatures. Fitting curves in (**c**–**f**) are for data points lower than the optimum temperature in substrate-saturated conditions (*T*_opt-sat_, ~26 °C for station J1; ~29 °C for station JLR4). Solid lines in (**a**, **b**) and the white areas in (**c**–**f**) represent the Michaelis–Menten kinetics at temperatures lower than the *T*_opt-sat_. While dotted lines in (**a**, **b**) and the gray areas in (**c**–**f**) represent the Michaelis–Menten kinetics at temperatures greater than the *T*_opt-sat_. See Supplementary Table 2 for more details on the temperature dependence of *K*_m_ and *V*_max_. The data in (**a**) are presented as mean values, instead of standard deviation the given bars indicate the variation range of two independent experiments. Data in (**b**–**f**) are expressed as the mean values ± SD (*n* = 6 in (**b**); *n* = 10 in (**c**, **e**); *n* = 48 in (**d**, **f**); independent experiments).

To further explore how the substrate-regulated *T*_opt_ of AOR in marine environments, we designed a Michaelis–Menten (M–M) thermal kinetics experiment for J1 (substrate deprived sea basin) and JLR4 (substrate-replete upstream estuary) stations with distinctive substrate concentrations (see details in Methods). The experimental results revealed that at a given temperature, the responses of AOR along with substrate addition can be fitted by the classic M–M curve (Fig. 2a, b; Supplementary Table 2), i.e. the rate increased as the NH_4_^+^ concentration increased until the substrate became saturated. These M–M curves are temperature-dependent, with the maximum rate (*V*_max_) and the half-saturation constant (*K*_m_) increasing as the temperature increased until the saturation optimum temperature (*T*_opt-sat_) was reached (Fig. 2c–f). Note that the *T*_opt-sat_ was defined as the optimum temperature of *V*_max_ at saturated substrate level (*T*_opt-sat_, ~26 °C for station J1 and ~29 °C for JLR4 station; see Fig. 2c, d).

From the above results, we suggest that the *T*_opt-sat_ of a single-species derived from laboratory culture under a saturated substrate concentration^18^ may not properly represent its *T*_opt_ in the field, where ammonium is not always saturated. Some published biogeochemical models with a nitrification component have assumed that nitrification follows the Arrhenius relationship until temperature reaches the *T*_opt_^23,24^. However, the *T*_opt_ in these models is derived from pure laboratory cultures typically grown at saturating substrate concentrations (*T*_opt-sat_), which may cause an overestimation of rates in the warming ocean at ambient unsaturated substrate concentrations.

**Fig. 3:**
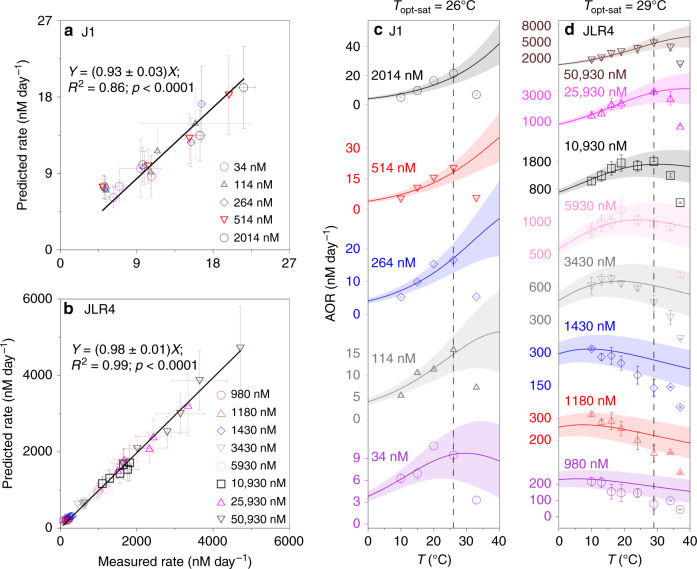
Validation plot for the rate predictions and observations. **a**, **b** Scatter plot of the predicted rates via the Dual Arrhenius and Michaelis–Menten kinetics model (DAMM model) and the measured rates under different substrate concentrations and temperatures (below the optimum temperature in substrate-saturated conditions, *T*_opt-sat_). Linear regressions between the model predictions and the measurements are presented (two-sided *t* test was used to generate the *p* value (95% confidence) to measure the strength of correlation coefficient. *p* values are uncorrected). **c**, **d** The rate patterns (dots) against temperature under different substrate concentrations. Curves stand for the predicted rates derived from the DAMM model and the symbols represent the measured rates. The shades denote the uncertainty of model prediction. The dashed black vertical lines represent the *T*_opt-sat_. The measured rates in (**a**, **c**) are presented as mean values, instead of standard deviation the given bars indicate the variation range of two independent experiments. The measured rates in (**b**, **d**) and the predicted rates in (**a**–**d**) are expressed as the mean values ± SD (*n* = 10 in (**a**, **c**); *n* = 48 in (**b**, **d**); independent experiments).

To examine the interactive effects of temperature and substrate on AOR and *T*_opt_, we adapted the Dual Arrhenius and Michaelis–Menten kinetics model (DAMM) developed by Davidson et al.^25,26^. The temperature sensitivity of *K*_m_ (Q10_Km_) and *V*_max_ (Q10_Vmax_) (Fig. 2c–f) was applied. In this model, the rate was determined by four parameters, i.e. substrate concentration, temperature, Q10_Km_, and Q10_Vmax_ (see Eq. (7) in Methods). By introducing the Q10_Vmax_ and Q10_Km_ values derived from station J1 and JLR4 into the DAMM model, we simulated how AOR responds to substrate and temperature synergistically (Fig. 3; Supplementary Fig. 2). Via the DAMM, we successfully predicted the AOR below the *T*_opt-sat_ under various ammonium levels (Fig. 3a, b). Moreover, we developed a *T*_opt_ model (see Eq. (9) in Methods) via the DAMM to see if we could predict the *T*_opt_ at various substrate levels. For the *T*_opt_ model, *T*_opt_ increased as substrate concentration increases under the criterion of Q10_Km_ > Q10_Vmax_ (Supplementary Fig. 2). This criterion was fulfilled in both J1 and JLR4 cases (Fig. 2c–f). We see the positive shift of *T*_opt_ due to the ammonium addition (up to ~100 nM at J1 station and up to ~10 μM at JLR4) can be closely predicted (Fig. 3c, d; Supplementary Fig. 2). Overall, the DAMM model successfully predicts the entire thermal response curve, including rates and *T*_opt_, except when the manipulated temperatures exceed *T*_opt-sat_ (Fig. 3; Supplementary Fig. 2). AOR drops significantly when temperature is greater than the *T*_opt-sat_, so heat-impaired biological enzyme activity^27,28^ might result in deviations from the relationship between *V*_max_ (*K*_m_) values and temperature from the Arrhenius law.

The substrate-dependent thermal optimum is attributable to the effect of temperature on biochemical kinetics and the structural stability of the enzymes. Increasing temperature promotes catalytic rate, thus, *V*_max_ increases due to increasing kinetic energy of reactants and rates of collision, as well as higher structural flexibility of enzymes^27,29^. However, higher structural flexibility (lower stability) also results in active sites with a reduced ability for ligand recognition and binding, therefore, lower kinetic efficiency. Accordingly, one important physiological response of an organism to rising temperature will be a reduction in substrate affinity (Supplementary Table 2), and thus higher substrate demands (i.e. higher *K*_m_ value)^25,29–31^. In other words, higher substrate levels help to compensate for enzyme structural stability losses and so promote growth rates at higher temperature. Note that some other microbes may respond differently to temperature, with Q10_Km_ ≤ Q10_Vmax_ for instance. This may lead to predictable yet unidirectional rate increases in response to warming (without substrate-regulated *T*_opt_) until the *T*_opt-sat_ is reached, regardless of substrate changes.

Similarly, nutrient-dependent *T*_opt_ has been reported for phytoplankton growth in pure cultures previously. For instance, Thomas et al.^32^ indicated that the *T*_opt_ for growth of a marine diatom was a saturating function of major nutrient (nitrate and phosphate) concentration, and that the *T*_opt_ could decrease by 3–6 °C at low concentrations relative to that at saturated nutrient levels. In addition to studies of pure cultures, field studies have also suggested that organisms may tolerate higher temperature stresses when nutrients are more abundant. For example, kelp (*Laminaria saccarina*) with high nitrogen reserves have more capacity for thermal adaptation^33^, while corals with symbionts limited by phosphate are more susceptible to heat-induced bleaching^34^. Although these examples are functionally and taxonomically distant from AOA and ammonia-oxidizing bacteria (AOB), strong similarities in substrate/nutrient regulation characteristics may imply a similar mechanism of enzymatic thermal responses between chemoautotrophs and photoautotrophs.

Nevertheless, the higher thermal optimum of AO in the estuarine system (e.g., JRL1, JLR2, and JLR3) than in the offshore environment (e.g., N3 and J1) can be explained by a substrate-regulated *T*_opt_. Note that field AOR represents explicitly the collective activity of the AO community composed of AOA and AOB, which may have distinctive thermal tolerances and affinities for substrate. Therefore, community structure very likely plays a role in modulating the thermal response patterns of community AOR in the field environments, in addition to substrate concentration.

### Rate proportion and community thermal optimum

**Fig. 4:**
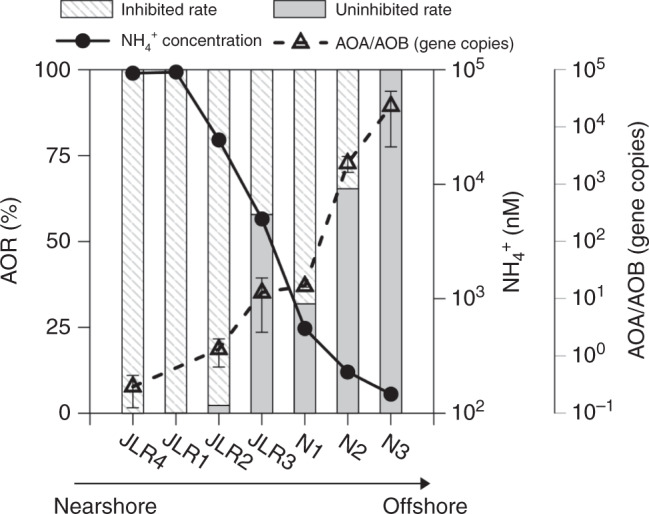
Rate inhibition along a substrate gradient. Proportional contributions of inhibited (slashed bars) and uninhibited (gray bars) ammonia oxidation rate by allylthiourea (ATU) to the bulk ammonia oxidation rate, the in situ ammonium concentration (solid line/filled circles) and the ratios of ammonia-oxidizing archaeal (AOA) vs. bacterial (AOB) *amo*A gene copy numbers (dashed line/open triangles). The gene copies data are expressed as the mean values ± SD (*n* = 3 independent experiments).

To further examine to what extent the community structure (proportions of AOA and AOB) might shape the thermal response patterns of community AOR observed in the field, we added allylthiourea (ATU) to inhibit the activity of AOB for rate discrimination (see Methods; Supplementary Discussions). Results showed that the inhibitory efficiency of AOR was gradually reduced with increasing offshore distance (Fig. 4). That is, from the estuary (JLR4, JLR1, JLR2, and JLR3) to the shelf (N1 and N2) and the sea basin (N3), the relative contribution of AOB to the community AOR dropped from as high as ~100% in the upper estuary down to ~70% in the shelf transition zone, and near 0% in the basin. Meanwhile, the AOA/AOB gene copies data (see Supplementary Methods) from estuary to sea basin (Fig. 4) also clearly show that the abundance of AOA relative to AOB increased exponentially with increasing offshore distance. A similar offshore pattern of community distribution was also observed in other regions, such as from the Pearl River estuary to the South China Sea^35^, and from the freshwater region of the Chesapeake Bay to the coastal and open ocean water column^36^. This pattern suggests that AOB strongly prefer substrate-replete niches, and vice versa for AOA^20,37^, agreeing well with our M–M experimental data that the substrate saturation condition for AOB-dominated water at JLR4 was several orders of magnitude higher than that for AOA-dominated water from J1 (Supplementary Table 2). The *K*_m_ values of AOB in JLR4 varied from 7 to 55 μM in accordance with varying temperatures from 10 to 37 °C, while the *K*_m_ values of AOA in J1 varied from 13 to 44 nM over a similar temperature range (Supplementary Table 2). Results were supportive of previous pure culture and field studies which showed the minimum ammonium demand for AOB is >1 μM and *K*_m_ values range from 28 to 4000 μM^38–41^, while minimum ammonium demand and *K*_m_ value for AOA are <10 and 133 nM^42^, respectively.

The low *K*_m_ for AOA indicates that the ambient substrate concentration can easily reach saturation state for AOA-dominated basin regions (see *K*_m_ values in Supplementary Table 2), thus, leading to a dome-shape thermal response pattern with the *T*_opt_ near to *T*_opt-sat_ likely around 26 °C (Figs. 1h, i, 3c), consistent with a report for an AOA isolate in lab culture^18^. Meanwhile, a *T*_opt-sat_ of ~30 °C (Figs. 1a, b, 3d) falls within the range of 29–35 °C reported for AOBs in pure culture^43,44^. Since the AOB-dominated JLR4 case showed a very wide *T*_opt_ as substrate varied, the negative response patterns at temperatures between 10 and 30 °C can be seen when the substrate was diluted below the several μM level, mimicking the seaward advection of a water mass and subsequent substrate dilution. Accordingly, we speculate that the three types of AOR thermal response patterns observed in the field were a result of the combined thermal response patterns of AOB and AOA along the substrate gradient. Although this type of negative response pattern has never been reported before in the field, we observed it at shelf stations and in our dilution experiment. This suggests that when AOBs dominate the community AOR in the shelf transition zone (Fig. 4), community AOR may respond negatively to temperature rise due to a low *T*_opt_ under substrate stress. In fact, comparison of the community AOR between ambient conditions (20 nM ^15^NH_4_^+^) and after significant tracer addition (2000 nM ^15^NH_4_^+^), we can clearly see the magnitude of rate stimulation at in situ temperature was higher at the shelf station dominated by AOB (M2 station, Fig. 1f) than at the basin station (J1 station, Fig. 1i) where the AOR was dominated by AOA. Such distinct rate stimulations by substrate additions implicitly indicate the degree of substrate saturation was lower on the shelf, where AOB contributed more to the AOR.

### Global change and future nitrification

Sea surface temperature (SST) will inevitably increase in most of the ocean as atmospheric greenhouse gas concentrations continue rising. According to the 2013 Intergovernmental Panel on Climate Change Representative Concentration Pathways (RCP) 8.5 scenario, mean global sea surface temperature (SST) will increase by up to 4 °C by the year 2100^45^, equivalent to a rise of 0.04 °C/y. The seasonal temperature (1 m depth) ranges from 13 to 32 °C for the Jiulong River estuary^46^ (Supplementary Fig. 1; Supplementary Table 3). The average SST rise over the period of 1960–2010 in the studied area was 0.02 °C/y^47^, about half of the predicted maximum increase rate of the global mean. On the other hand, hydrography of the northern South China Sea is influenced by regional climate and local hydrodynamics, including the western boundary current intrusion, internal waves and monsoon winds^48^. The water temperature of the upper 200 m in the northern South China Sea ranges from 13 to 31 °C at different depths^48,49^ and the mean temperature rise for the entire 200 m water column in the studied area was reported to be 0.09 °C/y from 1975 to 2005^50^. Generally speaking, the rise of water temperature in our study region is similar to or even larger than the global mean, suggesting this global warming rate can be applied in our study area (see Supplementary Discussion).

**Fig. 5:**
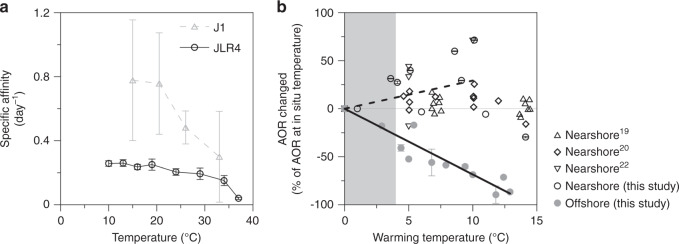
Thermal response projections in near- and offshore regions. **a** The thermal responses of specific affinity at the J1 and JLR4 stations. Data are expressed as the mean values ± SD (*n* = 10 in J1 station; *n* = 48 in JLR4 station; independent experiments). **b** Normalized warming-driven variations in ammonia oxidation rates. Rate changed (%) is relative to the ammonia oxidation rate (AOR) at in situ temperature. The mean increase (nearshore hollow dots) is denoted by the dashed line and the mean reduction (offshore, solid dots) is denoted by the solid black line. The shaded area represents the 4 °C increase in temperature mentioned in the IPCC study. Note that the stations where the surface salinities are lower than 32 are classified as nearshore station, and the others are classified as offshore stations. The data in (**b**) are presented as mean values, instead of standard deviation the given bars indicate the variation range of two independent experiments.

Using the thermal responses of kinetic parameters, we try to evaluate warming effects on the competition between AOB and AOA. The specific affinity (*α*), the ratio of *V*_max_/*K*_m_, can be used to represent a microbe’s ability to scavenge substrate from dilute environments. Thus, microorganisms with higher α values are superior competitors when substrate is limiting. We found the specific affinity was higher for AOA-dominated J1 than for AOB-dominated JLR4 (Supplementary Table 2; Fig. 5a). Moreover, α values of AOB vary in a narrower range relative to those of AOA as temperature changes. The less variable α may reflect that AOB cope better with variable temperature in their habitat (Supplementary Table 2; Fig. 5a). In contrast, AOA are more sensitive to temperature change. As aforementioned in the DAMM model, the criterion to have substrate-dependent *T*_opt_ is Q10_Km_ > Q10_Vmax_, which also results in a reduction in α during warming for both AOA and AOB. Yet, the relative reduction in AOA specific affinity as temperature increases is more significant (Fig. 5a), suggesting AOAs are more competitive in low temperature environments relative to AOBs, and so may not be favored in a warming ocean. On the other hand, the specific affinity of AOBs is insensitive to temperature change, suggesting their adaptation to nearshore environments with greater temperature fluctuation. The seaward gradient in temperature fluctuations and ammonium concentrations determine the nitrifier community, thus, thermal response pattern of community AOR observed in the field.

To predict the trends of AOR in different geographic spaces in warming ocean, we compile the available marine AOR data to examine the AOR changes empirically. If we assume the biogeographic distribution of AO community remains unchanged and consider solely the warming effect on AOR relative to the onsite temperature, we found the thermal responses of AOR in nearshore and offshore are quite different (Fig. 5b). More specifically, the higher *T*_opt_ of these AO communities in nearshore regimes allows ocean warming to promote coastal AOR when the temperature change increment is <10 °C. The AOR then drops with further temperature increases, likely due to the impaired enzyme activity as discussed above. On the other hand, community AOR decreases linearly with warming (Fig. 5b) in offshore waters, mainly because current ambient temperatures are close to *T*_opt-sat_ for AOAs and/or higher than *T*_opt_ of AOB in low substrate state.

According to the mean global sea surface temperature increase of 4 °C by 2100, AOR might increase by 0.4–30% in nearshore systems, with a mean increase of 13% (Fig. 5b). However, at contemporary substrate levels the community AOR in oligotrophic environments would decline by 13–33%, with a mean decrease of 27% in response to the 4 °C warming in ocean temperature (Fig. 5b).

Our results also suggest that the projected gradual increase in atmospheric deposition of anthropogenic nitrogen (including ammonium)^7,51,52^ may aid in the thermal adaption of ammonia-oxidizing microorganisms in ammonium-depleted offshore environments. However, most of the NH_4_^+^ from the atmosphere will be utilized first by phytoplankton in the surface layer above the nitracline, due to a competitive advantage of phytoplankton toward NH_4_^+^ relative to nitrifers^17,53^. If this ammonium was to be supplied directly to the niche of AOA, the offshore AOR may response positively to warming before reaching the *T*_opt-sat_. Note that AOAs prefer low temperature and cope with low substrate, so the peak AOR in open ocean generally appears near the nitracline^17^ where the vertical temperature gradient is also large. Therefore, the warming effect may also have a differential influence across the vertical scale.

In contrast, in the shallow nearshore dominated by AOBs additional inputs of anthropogenic NH_4_^+^ cannot further promote *T*_opt-sat_ to alter the empirical trend. Thus, a positive temperature response in nearshore regimes is more certain. According to our observational data (Fig. 4), although AOB gene copies are much less, AOBs appear to be the major AOR contributor in the mid-to-lower estuary, coastal seas and even in shelf zones unless ammonium is down to approximately hundreds of nM level. Thus, we speculate the niche space of AOBs may expand in the future in the land-ocean transition zone, because of their greater thermal adaptability and continuously increasing anthropogenic nitrogen inputs from continents.

The observed differential warming effects on eutrophic nearshore systems and oligotrophic offshore regions would have significant implications for climate feedbacks of the marine nitrogen cycle over a wide trophic range. For example, AOR increases in the coastal ocean would subsequently promote N_2_O emissions. In fact, field and laboratory culture studies showed that AOB have a greater potential than AOA to generate N_2_O. The N_2_O yield from AOB is approximately two times higher than that of AOA cultures^54^. Thus, in the nearshore where AOB-dominated AOR, N_2_O production may be further enhanced. This would serve as a positive feedback for global warming, while the suppression of AOR in offshore regions creates a negative feedback. On the other hand, during nitrogen recycling in turbid coastal and estuary systems, increasing AO would potentially enhance NO_2_^−^/NO_3_^−^ production to fuel denitrification in micro-niches^55^, which is another important pathway for N_2_O production. Thus, warming and excessive nitrogen input would further exacerbate the emissions of N_2_O from coastal seas.

In addition to climate feedbacks, the impact of global warming on oceanic ammonia oxidation may also change the distribution of nitrogen species. The suppression of AO by ocean acidification^8^ was also reported to further exacerbate the inhibition of AO caused by warming. At the base of the euphotic zone in the stratified open ocean, this dual suppression of AO will substantially reduce the amount of NH_4_^+^ converted back to NO_3_^−^ by nitrification, ultimately favoring smaller primary producers that are more competitive for NH_4_^+^ rather than NO_3_^−^. Meanwhile, NO_3_^−^-supported primary producers, such as large diatoms, would be at a competitive disadvantage. This might be disadvantageous for carbon export to the deep sea, and thus constitute a positive feedback to global warming.

Of course, multiple environmental factors such as acidification, stratification, deoxygenation, and UV light are changing simultaneously. To better predict how nitrification responds to interactive global change forcing, multiple factors should be included in experiments in the future. Moreover, both field and laboratory AO responses of the two steps of nitrification and associated N_2_O yield should also be investigated at various levels of ammonium to assess their impacts on global warming. Meanwhile, genomic and proteomic information is urgently needed to determine the physiological mechanisms of the species-specific temperature responses of marine AO organisms. Besides nitrification, to improve model predictive ability and have in-depth understanding of the biogeochemical role of nitrogen in microbial ecosystems, the temperature sensitivity of kinetic parameters of associated nitrogen processes needs to be investigated. Moreover, it is important to carefully examine the extrapolation from short-term experiments, such as our hourly manipulations, to long-term microbial responses to environmental change (decades). Analogous long-term experiments (thousands of generation) for marine nitrifiers are needed to explore the possibility of evolutionary adaptation to simultaneous changes in ammonium supplies and temperature in the future ocean.

## Methods

### Sampling

Samples were collected during five field cruises in 2016–2020. The sampling sites ranged from estuarine (the Jiulong River) to sea basin (the South China Sea) sites (Supplementary Fig. 1). Water samples were collected near the bottom of the euphotic zone where ammonia oxidation is the most active. The detailed sampling information, including sampling dates and depths, is reported in Supplementary Table 1. For the South China Sea cruises in May 2016, November 2016, and June 2017, the seawater samples were collected using a conductivity–temperature–depth (CTD) rosette fitted with 12-L Niskin bottles. Acid-washed high-density polyethylene (HDPE) bottles were used to collect the samples. A 100 mL portion of each nutrient sample from each station was analyzed for concentrations on-deck within hours of collection. At each sampling site, 5–10 L of water was collected for the manipulation experiments. During the Jiulong River cruise, samples were collected using a low-pressure electric bilge pump to gently draw water through acid-washed Tygon tubing into 10 L acid-washed polycarbonate (PC) bottles. After collection, these samples were stored in the dark and were transported to the shore-based laboratory within 6 h for the incubation experiments. For nutrient analyses, 100 mL of water was filtered (0.2 μm) and frozen at −20 °C in the laboratory.

### Chemical analyses

Salinity and temperature were measured using a CTD profiler. During the South China Sea cruises, the ammonium concentrations were measured immediately on-deck (except stations N1, N2, N3) using the fluorescence detection method^56^ with a detection limit of ~5 nM. The ammonium concentrations of the Jiulong River samples were measured using the indophenol blue spectrophotometric method^57^ with a detection limit of 0.6 μM. The nitrite and nitrate concentrations (NO_x_^−^) were measured using the chemiluminescence technique^58^ with a 0.01 μM detection limit.

### Ammonia oxidation rate incubation experiments

^15^N-labeled ^15^NH_4_Cl (98 atom% ^15^N; Sigma-Aldrich, 299251-1G, Lot#TA2540V) was added to the water samples to determine the ammonia oxidation rates. The final concentrations of ^15^NH_4_Cl are reported in Fig. 1.

The South China Sea and Jiulong River samples were incubated in the dark (1–2 replicates) for ~24 h and ~3 h, respectively. Detailed information about this method has been presented in previous studies^59,60^. The control samples were filtered immediately (*t*_0_) after tracer addition. All of the incubation procedures were terminated by filtering samples through a 0.22 μm polycarbonate membrane, and the filtrate was frozen at −20 °C until analysis. To examine the linearity of the ^15^NO_x_^−^ production within the incubation period, we carried out time series incubation experiments for samples from both estuary and sea basin stations (Supplementary Fig. 1 and Supplementary Fig. 3). Highly correlated linear regressions for time series incubations suggest the isotope dilution effect and community changes were minimal within the incubation periods.

The final added tracer concentration of <2 times the in situ ammonium concentration was defined as the ammonia oxidation rates for the ambient substrate level (AOR_ambient_). Higher additions were defined as the ammonia oxidation rates at the enriched substrate level (AOR_enriched_).

### Temperature manipulation experiments

To quantify the effect of temperature on AO, we applied a gradient of 4–5 temperatures ranging from ~14 to ~34 °C at ~5 °C intervals (Fig. 1). The temperature was controlled by a thermostat-controlled incubator with a precision of ±1 °C.

### Michaelis–Menten kinetics experiments

We conducted experiments to determine the Michaelis–Menten kinetics of ammonia oxidation in response to temperature. For the basin area where NH_4_^+^ concentration was low, such as station J1, we added substrate directly to obtain M–M curves under manipulated temperatures. Varied ammonium concentrations were recorded for water samples incubated for ~24 h at different temperatures (Fig. 2a). In areas where AOB dominated, such as in JLR4 estuary, the concentration of substrate was over the saturation concentration of AOB (dozens of μM levels). To test how field AOB respond to temperature change in low substrate and to obtain the M–M kinetic parameters of AOB collected from the field, we tried to dilute the ammonium in the freshwater water sample collected from the very upper estuary. The procedure is as follows: (1) we collected microbes from 15-L seawater by using 0.2 μm PC membrane, which might enrich the ammonia-oxidizing microorganisms, and then (2) all microbes on the membrane were washed into the mixture of 50 mL in situ seawater and 5 L Milli-Q water (diluted 100 times), (3) a wide gradient of NH_4_^+^ concentration was added afterward for M–M kinetics experiments under manipulated temperatures (Fig. 2b).

### Inhibition experiments

In an attempt to distinguish the relative contributions of archaeal and bacterial ammonia oxidation, parallel incubations with ^15^NH_4_^+^ and 80 μM allylthiourea (ATU) were conducted at station JLR1, JLR2, JLR3, JLR4, N1, N2, and N3 (Fig. 4). ATU was utilized to inhibit *β*-proteobacterial AOB with minimal effects on AOA at the concentration in our incubations (~80 μM)^61^. Thus, rates of ammonia oxidation in samples tested with ATU are the rates of AOA obtained by suppressing AOB. The rates of AOB were then derived by difference from the control without ATU addition.

### Measurement of ammonia oxidation rate

The δ^15^N of the NO_x_^−^, end product of ammonia oxidation, was determined using the denitrifier method^62,63^. The isotopic composition of the bacterially-produced N_2_O was measured using a Gasbench-II (Thermo Fisher) connected to an isotope ratio mass spectrometer (Thermo Delta V Advantage). In order to obtain accurate δ^15^N values, three NO_3_^−^ international reference materials (δ^15^N_USGS 34_ = −1.80‰, δ^15^N_IAEA N3_ = 4.70‰, and δ^15^N_USGS 32_ = 180.00‰) were used to calibrate the δ^15^N–NO_x_^−^ of the samples. The detection limit was an absolute amount of 4 nmol N, and the precision of δ^15^N was 0.2‰.

The rate was calculated using Eq. (1)^16,64^:

1 $${\mathrm{AOR}} = \frac{{\Delta {\mathrm{[}}{}^{15}{\mathrm{NO}}_{\mathrm{X}}^{\mathrm{ - }}{\mathrm{]}}}}{{{\mathrm{f}}_{{}^{15}{\mathrm{NH}}_{\mathrm{4}}^{\mathrm{ + }}}\times t}}{\mathrm{,}}$$

where Δ[^15^NO_x_^−^] is the change in concentration of ^15^NO_x_^−^ between the start and the end of the incubation due to ammonia oxidation; f^15^_NH4_^+^ is the fraction of ^15^NH_4_^+^ labeled with ^15^N at the start of the incubation; and *t* is the length of incubation.

Note that the incubation experiments were conducted in different cruises. During one of the cruises, we did not have sufficient manpower to do on-deck NH_4_^+^ measurements (marked by * for stations N1, N2, and N3 in Fig. 1). The NH_4_^+^ concentrations determined might be higher in lab than on-deck measurements. The influence will be a significant overestimate in AOR_ambient_ but insignificant overestimate in AOR_enriched_. Thus the response patterns were utilized for discussion while the substrate enrichment effect is not discussed.

### The DAMM model and *T*_opt_ model

The Dual Arrhenius and Michaelis–Menten kinetics model (DAMM) was initially introduced by Davidson et al.^25,26^. The initial model combined the Michaelis–Menten (Eq. (2)) and temperature sensitivities of maximum rate (*V*_max_, Eq. (3)) and half-saturation constant (*K*_m_, Eq. (4))

2 $${\mathrm{AOR}} = \frac{{V_{{\mathrm{max}}}C}}{{K_{\mathrm{m}} + C}}{\mathrm{,}}$$

where AOR is the ammonia oxidation rate; *V*_max_ is the maximum ammonia oxidation rate in substrate-saturated conditions; *K*_m_ is the half-saturation constant corresponding to the concentration of substrate at which the rate is 50% of *V*_max_, and measures performance at low substrate concentrations. *C* is the substrate concentration.

Temperature dependence of *V*_max_ should follow a left-skewed unimodal function of temperature. Within the optimum temperature in substrate-saturated conditions (*T*_opt-sat_), *V*_max_ is expected to increase exponentially with temperature^65^, while the temperature dependence of *K*_m_ is inconsistent^66–68^. Several experimental studies in algae, plants and bacteria found positive correlations^66^ between *K*_m_ and temperature, while others found a negative relationship^67^ and still others found no evidence of temperature dependence^68^. According to theoretical study, *K*_m_ is expected to increase with temperature^32^. We assumed *K*_m_ to have a similar thermal response to *V*_max_, with the temperature dependence within the *T*_opt-sat_ of both *V*_max_ and *K*_m_ following the Boltzmann–Arrhenius equation.

3 $$V_{{\mathrm{max}}} = V_{{\mathrm{max0}}}{\mathrm{e}}^{b_{{\mathrm{Vmax}}}T}{\mathrm{,}}$$

4 $$K_{\mathrm{m}} = K_{{\mathrm{m0}}}{\mathrm{e}}^{b_{{\mathrm{Km}}}T}{\mathrm{,}}$$

where *V*_max_ and *K*_m_ are the maximum rate and the half-saturation constant, respectively, at temperature (*T*); *V*_max0_ and *K*_m0_ are the maximum rate and the half-saturation constant, respectively, at 0 °C; *b*_Vmax_ and *b*_Km_ are held constant and represent the temperature sensitivity coefficient.

The temperature sensitivities of *V*_max_ (Q10_Vmax_) and *K*_m_ (Q10_Km_), which reflect the ratios of the change in *V*_max_ and *K*_m_ in response to a temperature increase of 10 °C, were calculated using Eqs. (5) and (6).

5 $${\mathrm{Q10}}_{{\mathrm{Vmax}}} = {\mathrm{e}}^{{\mathrm{10b}}_{{\mathrm{Vmax}}}}{\mathrm{,}}$$

6 $${\mathrm{Q10}}_{{\mathrm{Km}}} = {\mathrm{e}}^{{\mathrm{10b}}_{{\mathrm{Km}}}}{\mathrm{.}}$$

The DAMM model combines the Michaelis–Menten kinetic equation (Eq. 2) with the temperature dependence of *V*_max_ and *K*_m_ (Eqs. (3)–(6)) in order to describe how the substrate concentration and temperature affect the rate together (Eq. 7).

7 $${\mathrm{AOR}} = \frac{{V_{{\mathrm{max0}}} \times {\mathrm{(Q10}}_{{\mathrm{Vmax}}}{\mathrm{)}}^{T/10} \times C}}{{K_{{\mathrm{m0}}} \times {\mathrm{(Q10}}_{{\mathrm{Km}}}{\mathrm{)}}^{T/10} + C}}{\mathrm{,}}$$

where AOR is the ammonia oxidation rate at temperature *T*; *V*_max0_ and *K*_m0_ are the *V*_max_ and *K*_m_ at 0 °C, respectively; Q10_Vmax_ and Q10_Km_ are the ratios of the change in *V*_max_ and *K*_m_, respectively, as a consequence of a 10 °C increase in temperature (i.e. the temperature sensitivity coefficient); and *C* is the substrate concentration.

The DAMM model has been shown to successfully predict observations of microbes in soil systems for a range of substrate concentrations and incubation temperatures in terms of laboratory enzyme assays of *β*-glucosidase and phenol-oxidase^26^.

In fact, since *T*_opt_ is defined as the temperature at which the rate reaches its maximum value at a fixed substrate concentration, the *T*_opt_ can be determined when the first-order derivative of the relationship between the rate and temperature is equal to 0 (the inflection point). Therefore,

8 $$\frac{{\partial _V}}{{\partial _T}} = {\mathrm{0}}{\mathrm{.}}$$

When combined with Eqs. (7) and (8) this results in the *T*_opt_ equation:

9 $$T_{{\mathrm{opt}}} = \frac{{{\mathrm{10}} \times {\mathrm{log}}\left[ {\frac{{C \times {\mathrm{logQ10}}_{{\mathrm{Vmax}}}}}{{K_{{\mathrm{m0}}}\left( {{\mathrm{logQ10}}_{{\mathrm{Km}}} - {\mathrm{logQ10}}_{{\mathrm{Vmax}}}} \right)}}} \right]}}{{{\mathrm{logQ10}}_{{\mathrm{Km}}}}}{\mathrm{.}}$$

### Statistics and reproducibility

We analyzed our results with the collected data using the commercial software, the Grapher (version 15, Golden Software) and SPSS (IBM, version 19). The phenomenon of substrate-regulated thermal optimum for ammonia oxidation rate was reproduced at five independent stations.

### Reporting summary

Further information on research design is available in the Nature Research Reporting Summary linked to this article.

## Supplementary information

Supplementary Information

Peer Review File

Reporting Summary

## Data Availability

The datasets generated or analyzed during the current study are available in the Figshare repository, 10.6084/m9.figshare.12187794.v1.
