# Supplementary material for: Substrate regulation leads to differential responses of microbial ammonia-oxidizing communities to ocean warming
Source: Nat Commun. 2020 Jul 14;11:3511. doi: 10.1038/s41467-020-17366-3 (PMC7360760; doi:10.1038/s41467-020-17366-3)
Supplement: Supplementary file 1 — Supplementary Information [file 41467_2020_17366_MOESM1_ESM.pdf]

Supplementary Information

Substrate regulation leads to differential responses of microbial ammonia-oxidizing communities to ocean warming

Zheng et al.

Supplementary Information

Substrate regulation leads to differential responses of microbial ammonia-oxidizing communities to ocean warming

Zhen-Zhen Zheng[1], Li-Wei Zheng[2], Min Nina Xu[1,3], Ehui Tan[2], David A. Hutchins[3], Wenchao Deng[1], Yao Zhang[1], Dalin Shi[1], Minhan Dai[2] and Shuh-Ji Kao[1, 2*]

[1] State Key Laboratory of Marine Environmental Science, College of the Environment & Ecology, Xiamen University, Xiamen, Fujian, P. R. China.

[2] State Key Laboratory of Marine Environmental Science, College of Ocean and Earth Sciences, Xiamen University, Xiamen, Fujian, P. R. China.

[3] Department of Biological Sciences, University of Southern California, Los Angeles, CA, USA

[*] Correspondence should be addressed to sjkao@xmu.edu.cn

Supplemental Methods

Quantification of Archaeal and Bacterial *amo*A Genes

For *amoA* gene abundance, one liter seawater sample was filtered through a 47 mm 0.22 μm polycarbonate filter immediately after sampling, and the folded filter was wrapped and stored in an ice chest with dry ice and was transferred into a -80°C freezer in the laboratory until further gene analysis. DNA was extracted using the UltraClean Soil DNA kit (MoBio, San Diego, CA, USA) following the manufacturer's instructions. Archaeal and *β*-proteobacterial *amoA* gene sequences were amplified using primer sets Arch-amoAF and Arch-amoAR[1], amoA-34F and amoA-2R[2], respectively.

The polymerase chain reaction (PCR) mixture was prepared in accordance with *Hu et al.*[3]. The PCR conditions of archaeal and *β*-proteobacterial *amoA* gene were applied as described by *Francis et al.*[1] and *Kim et al.* [2], respectively. Standard curves were created using ten-fold dilution series of each DNA standard ranging 10^0 to 10^9 gene copies L^{-1} for the archaeal and *β*-proteobacterial *amoA* gene. Standards, samples and non-template controls were amplified in triplicate with each primer set.

qPCR amplification was carried out as described previously[4,5] with slight modifications. The qPCR reaction mixture (25 μL) contained 12.5 μL SYBR® Premix

Ex Taq™ II (TakaRa, Dalian, China), 5 μg Bovine Serum Albumin (BSA), 0.4 μM of each primer and 1 μL template DNA of 1-10 ng. Thermal cycling for archaeal *amoA* gene consisted of initial denaturation at 95 °C for 30 s followed by 40 cycles of denaturation at 95 °C for 30 s, primer annealing at 53 °C for 60 s, and extension at 72 °C for 45 s. Thermal cycling for *β*-proteobacterial *amoA* gene consisted of initial denaturation at 94 °C for 15 seconds followed by 50 cycles of denaturation at 94 °C for 15 seconds, primer annealing at 60 °C for 30 seconds, and extension at 72 °C for 1.5 min, with a final extension of 1 second at 78 °C to assure stringent product detection. The amplification efficiencies of PCR of archaeal and *β*-proteobacterial *amoA* genes were always between 85 and 96% with R^2 values >0.99. The specificity of the qPCR reactions was confirmed by melting curve analysis, agarose gel electrophoresis.

Supplemental Discussions

Responses of the ammonia oxidation bacteria and archaea to inhibitor

In pure culture and field culture experiments, allylthiourea (ATU) is often used to inhibit the ammonia oxidation (AO) process. ATU can complex with Cu^{2+} required by NH_3 monooxygenase (AMO) during ammoxidation to inhibit the AO process. Previous studies showed that both AOA and AOB contain AMO [6,7], but AOA and AOB have different sensitivity to ATU. Shen et al.,[8] studied the responses of the terrestrial AOA and AOB to nitrification inhibitors and found that AOB activity was inhibited by 80% when ATU concentration was less than 1 μM, while the rate of ammonia oxidation was only slightly different from that of the control group when the ATU concentration was 80 μM. Martens-Habbena et al.,[9] tested the responses of AOA and AOB strains to ATU. It found that *Candidatus Nitrosopumilus maritimus* (SCM1) was active at an ATU concentration of 10 μM, and its rate was inhibited by 50% at 100 μM ATU, while ~ 97% inhibited at 330 μM ATU. Compared to SCM1, HCA1 isolated from the Hood Canal (P10 station) is much less sensitive to ATU. It showed no apparent inhibition up to 33 μM ATU, only ~ 14% inhibition at 100 μM ATU, and still retained nearly 40% of its maximum growth rate at 330 μM ATU. In contrast, all

tested AOB strains were completely inhibited by less than 100 μM ATU[9]. Study in the Gulf of California found that the activity of AOA was barely inhibited after 24 h of addition of 86 μM ATU[10]. Therefore, we chose a dose of ATU of 80 μM to distinguish the contribution of AOA and AOB to the rate.

Manipulated temperature and temperature variation

The manipulated temperature range set (~14 - ~34°C) was comparable with the natural seasonal temperature variation (13 – 32 °C in estuary and 13 - 31°C in sea basin) in marine AO microbial habitats (Supplementary Table 3). Meanwhile, at an increase rate of 0.02 °C (nearshore) or 0.09 °C (offshore) per year[11,12], the temperature in 2100 will be ~2 °C (nearshore) and ~7 °C (offshore) higher than today, thus, the temperature range set by this study was also comparable to the temperature range of study regions expected in 2100 year (~15 - ~34°C in estuary and ~20 – ~38 °C in sea basin).

Supplementary tables

Supplementary Table 1 List of experimental parameters including sampling dates, stations, sampling depths, temperature, salinity, ambient nitrogen concentration

Date	Station	Sampling Depth (m)	Bottom Depth(m)	Temperature (°C)	Salinity (psu)	NH_4^+ (nM)	NO_x^- (nM)
2017.4.19	JLR1	1.0	5.0	25.4	1.5	95500	211300
2017.4.19	JLR2	1.0	6.0	23.9	14.2	24450	123000
2017.4.19	JLR3	1.0	11.2	19.9	30.3	5000	16000
2020.1.8	JLR4	0.5	1.1	26.2	0.0	93000	280000
2016.11.30	N1	25	28	23.0	31.1	550	4192
2016.5.18	M1	60	75	21.0	34.6	104	2530
2016.11.13	N2	60	737	24.6	34.4	230	3690
2016.5.19	M2	100	765	19.2	34.7	45	8282
2016.11.29	N3	100	2671	21.1	34.6	147	3868
2017.6.24	J1	100	4079	20.6	34.6	14	10190

Supplementary Table 2 The Michaelis–Menten half-saturation constant (K_m), maximum rate (V_{max}) and specific affinity (α) of stations J1 and JLR4 at different manipulated temperatures

Station	Temperature (°C)	K_m (nM)	V_{max} (nM d^{-1})	α (d^{-1})	R^2	n
J1	15	13.5 ± 6.5	10.5 ± 0.6	0.78 ± 0.38	0.72	10
	20	21.0 ± 8.7	15.9 ± 0.9	0.76 ± 0.32	0.78	
	26	44.1 ± 9.2	21.3 ± 0.9	0.48 ± 0.10	0.96	
	33	22.0 ± 20.6	6.6 ± 0.9	0.30 ± 0.28	0.48	
JLR4	10	7327 ± 467	1885 ± 42	0.26 ± 0.02	1.00	48
	13	8961 ± 681	2333 ± 64	0.26 ± 0.02	1.00	
	16	15677 ± 993	3697 ± 98	0.24 ± 0.02	1.00	
	19	16459 ± 2095	4129 ± 222	0.25 ± 0.03	0.99	
	24	27454 ± 2479	5625 ± 246	0.20 ± 0.02	1.00	
	29	47585 ±7604	9208 ± 865	0.19 ± 0.04	1.00	
	34	54907 ± 9744	8305 ± 905	0.15 ± 0.03	1.00	
	37	39518 ± 5606	1561 ± 123	0.04 ± 0.01	1.00	

Supplementary Table 3 Temperature records for Stations ESTR and SEATS

Station	Date	Depth (m)	Temperature (°C)
ESTR (Estuary)	2015.12.16	1.0	19
	2016.9.5	1.0	32
	2017.4.19	1.0	25
	2017.11.27	1.0	21
	1990-1995	2.0	13 – 32[13]
SEATS (sea basin)	2014.6 – 2016.8	10	22 – 31[14]
	2014.6 – 2016.8	100	16 - 25[14]
	2014.6 – 2016.8	200	13 - 17[14]
	2018.1.28	100	17
	2018.8.5	100	19

Supplementary figures

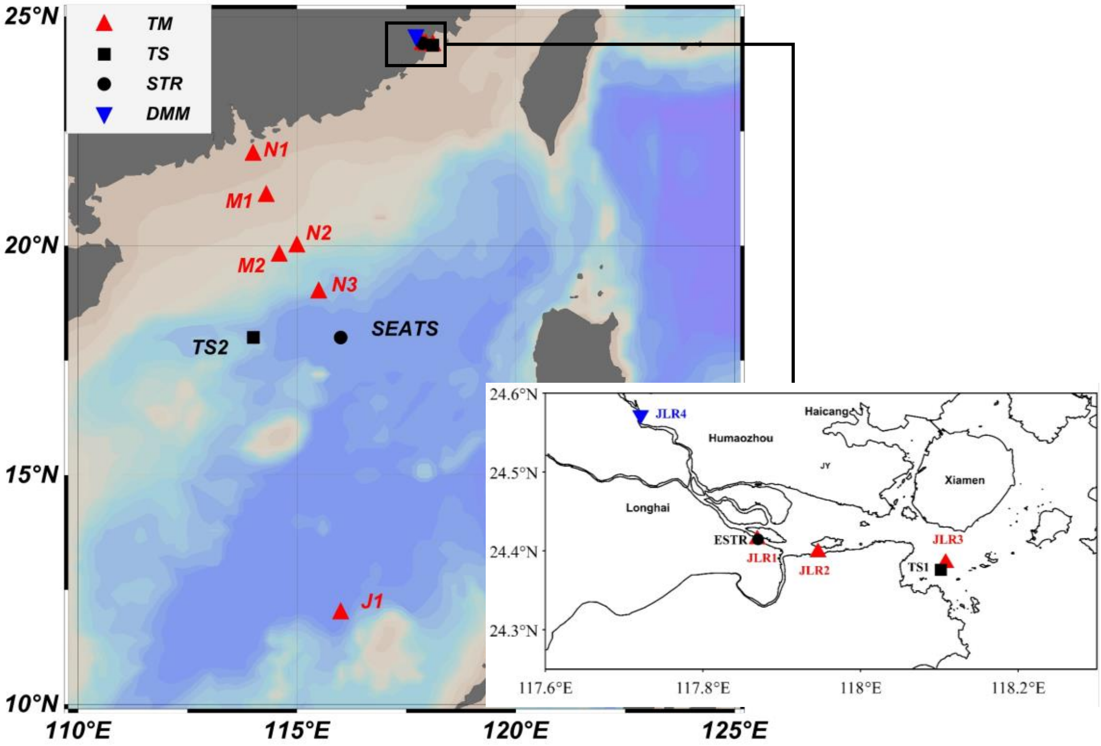

Supplementary Fig. 1 Locations of temperature manipulation (TM, red triangles) experiments of the ammonia oxidation rate (AOR), AOR time series incubation experiments (TS, blank squares), and the seasonal temperature records (STR, blank dots). The Michaelis–Menten kinetics experiment of AOR from JLR4 was conducted after the nutrient dilution (DMM, blue inverted triangle; see Methods). Sample IDs of M, N, and J represent collections in May 2016, November 2016, and June 2017, respectively. Sample IDs of JLR represent collections in the Jiulong River.

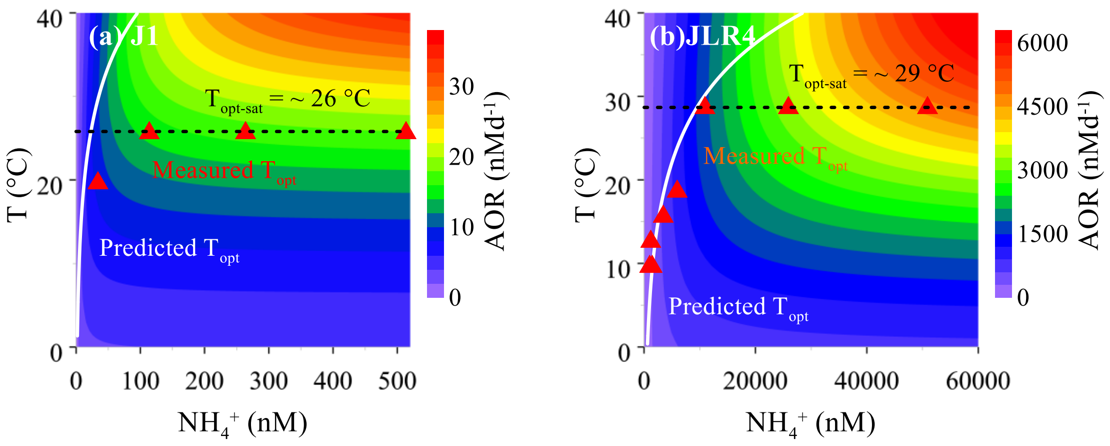

Supplementary Fig. 2 The joint effects of temperature and ammonium concentration on ammonia oxidation rate based on the DAMM model (equation 7) for AOA-dominated J1 station and AOB-dominated JLR4 station. The white solid lines represent the predicted optimum temperature (T_{opt}) based on T_{opt} model (equation 9). The red triangles represent the measured T_{opt} under varying ammonium concentration. The black dash lines represent the optimum temperature in substrate-saturated conditions ($T_{opt\text{-}sat}$)

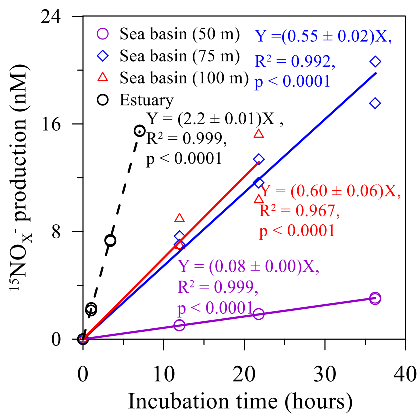

Supplementary Fig. 3 Examples of the time series incubations. $^{15}NO_x^-$ produced is plotted against incubation time for estuary TS1 station (black circle and dashed line) and sea basin TS2 station (red, blue and purple symbols and solid lines). (n =2 independent experiments). Regression (Two-sided t test was used to generate the p value (95% confidence) to measure the strength of correlation coefficient. p values are uncorrected) parameters are shown in corresponding colors.

Supplementary References

1. Francis, C. A., Roberts, K. J., Beman, J. M., Santoro, A. E. & Oakley, B. B. Ubiquity and diversity of ammonia-oxidizing archaea in water columns and sediments of the ocean. *Proc. Natl. Acad. Sci. USA* **102**, 14683-14688 (2005).

2. Kim, O. S., Junier, P., Imhoff, J. F. & Witzel, K. P. Comparative analysis of ammonia monooxygenase (*amo*A) genes in the water column and sediment–water interface of two lakes and the Baltic Sea. *FEMS microbial. ecol*., **66**: 367-378 (2008).

3. Hu, A. et al. Community structures of ammonia-oxidising archaea and bacteria in high-altitude lakes on the Tibetan Plateau. *Freshwat. Biol.* **55**, 2375–2390 (2010).

4. Mincer, T. J. et al. Quantitative distribution of presumptive archaeal and bacterial nitrifiers in Monterey Bay and the North Pacific Subtropical Gyre. *Environ. Microbiol.* **9**, 1162–1175 (2007).

5. Hu, A., Jiao, N. & Zhang, C. L. Community structure and function of planktonic Crenarchaeota: changes with depth in the South China Sea. *Microb. Ecol*. **62**, 549-563 (2011).

6. Walker, C. et al. *Nitrosopumilus maritimus* genome reveals unique mechanisms for nitrification and autotrophy in globally distributed marine crenarchaea. *Proc. Natl. Acad. Sci. USA* **107**, 8818-8823 (2010).

7. Zahn, J. A., Arciero, D. M., Hooper, A. B. & Dispirito, A. A. Evidence for an iron center in the ammonia monooxygenase from *Nitrosomonas europaea*. *FEBS Lett*. **397**, 35-38 (1996).

8. Shen, T., Stieglmeier, M., Dai, J., Urich, T. & Schleper, C. Responses of the terrestrial ammonia-oxidizing archaeon Ca. *Nitrososphaera viennensis* and the ammonia-oxidizing bacterium *Nitrosospira multiformi*s to nitrification inhibitors. *FEMS Microbiol. Lett.* **344**, 121-129 (2013).

9. Martens-Habbena, W. et al. The production of nitric oxide by marine ammonia-oxidizing archaea and inhibition of archaeal ammonia oxidation by a nitric oxide scavenger. *Environ. Microbiol.* **17**, 2261-2274 (2015).

10. Santoro, A. E. & Casciotti, K. L. Enrichment and characterization of ammonia-oxidizing archaea from the open ocean: phylogeny, physiology and stable isotope fractionation. *ISME J.* **5**, 1796–1808, (2011).

11. Yan, X., Cai, R. & Bai, Y. Long-term change of the marine environment and plankton in the Xiamen Sea under the influence of climate change and human sewage. *Toxicol. Environ. Chem.* **98**,

669-678 (2016).

12. Ning, X. et al. Long term changes in the ecosystem in the northern South China Sea during 1976-2004. *Biogeosciences* **6,** 2227-2243 (2009).

13. Yang Y. and Hu M. Biogeochemical research in the Jiulong River estuary. In: *Biogeochemical Research in Main Estuaries in China* (eds Zhang, J.). Chinese Ocean Press (1996).

14. Xing, X., Qiu, G., Boss, E., and Wang, H. Temporal and vertical variations of particulate and dissolved optical properties in the South China Sea. *J. Geophys. Res-Oceans*, **124**, 2019.
