# Supplementary material for: Substrate regulation leads to differential responses of microbial ammonia-oxidizing communities to ocean warming
Source: Nat Commun. 2020 Jul 14;11:3511. doi: 10.1038/s41467-020-17366-3 (PMC7360760; doi:10.1038/s41467-020-17366-3)
Supplement: Supplementary file 3 — Reporting Summary [file 41467_2020_17366_MOESM3_ESM.pdf]

nature research

Corresponding author(s): Shuh-Ji Kao

Last updated by author(s): Jun 24, 2020

Reporting Summary

Nature Research wishes to improve the reproducibility of the work that we publish. This form provides structure for consistency and transparency in reporting. For further information on Nature Research policies, see our Editorial Policies and the Editorial Policy Checklist.

Statistics

For all statistical analyses, confirm that the following items are present in the figure legend, table legend, main text, or Methods section.

n/a	Confirmed	
☐	☒	The exact sample size (n) for each experimental group/condition, given as a discrete number and unit of measurement
☐	☒	A statement on whether measurements were taken from distinct samples or whether the same sample was measured repeatedly
☐	☒	The statistical test(s) used AND whether they are one- or two-sided<br>*Only common tests should be described solely by name; describe more complex techniques in the Methods section.*
☐	☒	A description of all covariates tested
☐	☒	A description of any assumptions or corrections, such as tests of normality and adjustment for multiple comparisons
☐	☒	A full description of the statistical parameters including central tendency (e.g. means) or other basic estimates (e.g. regression coefficient) AND variation (e.g. standard deviation) or associated estimates of uncertainty (e.g. confidence intervals)
☐	☒	For null hypothesis testing, the test statistic (e.g. F, t, r) with confidence intervals, effect sizes, degrees of freedom and P value noted<br>*Give P values as exact values whenever suitable.*
☒	☐	For Bayesian analysis, information on the choice of priors and Markov chain Monte Carlo settings
☐	☒	For hierarchical and complex designs, identification of the appropriate level for tests and full reporting of outcomes
☐	☒	Estimates of effect sizes (e.g. Cohen's d, Pearson's r), indicating how they were calculated

Our web collection on statistics for biologists contains articles on many of the points above.

Software and code

Policy information about availability of computer code

Data collection	We collected our data from the published literatures. No software was used.
Data analysis	We analyzed our results with the collected data using the commercial software, the Grapher (version 15, Golden Software) and SPSS (IBM, version 19).

For manuscripts utilizing custom algorithms or software that are central to the research but not yet described in published literature, software must be made available to editors and reviewers. We strongly encourage code deposition in a community repository (e.g. GitHub). See the Nature Research guidelines for submitting code & software for further information.

Data

Policy information about availability of data

All manuscripts must include a data availability statement. This statement should provide the following information, where applicable:

- Accession codes, unique identifiers, or web links for publicly available datasets
- A list of figures that have associated raw data
- A description of any restrictions on data availability

The datasets generated during and/or analysed during the current study are available in the Figshare repository, https://doi.org/10.6084/m9.figshare.12187794.v1.

Field-specific reporting

Please select the one below that is the best fit for your research. If you are not sure, read the appropriate sections before making your selection.

☐ Life sciences ☐ Behavioural & social sciences ☒ Ecological, evolutionary & environmental sciences

For a reference copy of the document with all sections, see nature.com/documents/nr-reporting-summary-flat.pdf

Ecological, evolutionary & environmental sciences study design

All studies must disclose on these points even when the disclosure is negative.

Study description	This study mainly focused on the effects of temperature and substrate concentration on ocean ammonia oxidation rates. This experiment includes 4 - 8 temperature ranging from ~10 to ~37°C at ~5°C intervals at each sampling site and 2 - 8 substrate concentration gradients at substrate manipulation incubations. 1-6 replicates for each experimental set.
Research sample	The water samples were collected from coastal eutrophic waters to offshore oligotrophic regions based on substrate (ammonium) concentration gradient to investigate the temperature sensitivity of ammonia oxidation rate under different substrate levels.
Sampling strategy	Water samples were collected near the bottom of the euphotic zone where ammonia oxidation is the most active. 5 - 15 L bottles were used to collect the samples. 15 mL for rate incubation × 6 parallels × 8 temperature gradients × 8 substrate concentration gradients = 5.8 L
Data collection	The rate-related data was measured using a Thermo Finnigan Delta V Plus isotope ratio mass spectrometer and the data recorded by Zheng, Z. Z.
Timing and spatial scale	Samples were collected during five field cruises in 2016 – 2020 (2016.5; 2016.11; 2017.4; 2017.6; 2020.1). The sampling sites including estuarine regions, shelf, and a sea basin. All of the sampling sites were included in an area with a range of latitude from 11 - 24.6°N and a range of longitude from 114.5 - 118.2°E. The timing and location of sample collection choices was based on the season (temperature) and substrate (ammonium) concentration gradient.
Data exclusions	No data was excluded from the analysis in this study.
Reproducibility	The phenomenon of substrate-regulated thermal optimum for ammonia oxidation rate was reproduced at 5 independent stations.
Randomization	The sample collected from one site were mixed then allocated into different groups for manipulated experiment randomly.
Blinding	We collected the historical data on the temperature dependence of ammonia oxidation processes , so blinding was not possible.

Did the study involve field work? ☒ Yes ☐ No

Field work, collection and transport

Field conditions	The air temperature during field sampling was 16 - 32°C.
Location	The samples were collected from estuary (water depth: 1-11m; latitude:24.3-24.6°N longitude:117.7-118.1°E), shelf(water depth: 28-760m; latitude:19-23°N longitude:114-115°E) and sea basin (water depth: 2600-4000m; latitude: 10-20°N longitude:115-117°E)
Access & import/export	The estuary water samples were stored in-dark in the field and transported back to laboratory within 6 h for chemical analyses and incubation experiments. While the water samples in offshore stations were incubated on-board then frozen at -20°C and transported back to laboratory for chemical analyses. Our permits were obtained from Xiamen University on Sep. 5, 2016.
Disturbance	Nearly no disturbance was caused by the study since only 5 - 15 L seawater were collected from each site.

Reporting for specific materials, systems and methods

We require information from authors about some types of materials, experimental systems and methods used in many studies. Here, indicate whether each material, system or method listed is relevant to your study. If you are not sure if a list item applies to your research, read the appropriate section before selecting a response.

Materials & experimental systems

n/a	Involved in the study
☒	☐ Antibodies
☒	☐ Eukaryotic cell lines
☒	☐ Palaeontology and archaeology
☒	☐ Animals and other organisms
☒	☐ Human research participants
☒	☐ Clinical data
☒	☐ Dual use research of concern

Methods

n/a	Involved in the study
☒	☐ ChIP-seq
☒	☐ Flow cytometry
☒	☐ MRI-based neuroimaging
