# Supplementary material for: Substrate regulation leads to differential responses of microbial ammonia-oxidizing communities to ocean warming
Source: Nat Commun. 2020 Jul 14;11:3511. doi: 10.1038/s41467-020-17366-3 (PMC7360760; doi:10.1038/s41467-020-17366-3)
Supplement: Supplementary file 2 — Peer Review File [file 41467_2020_17366_MOESM2_ESM.docx]

Peer Review Information

**Journal:** Nature Communications

**Manuscript title:** Substrate regulation leads to differential responses of microbial ammonia-oxidizing communities to ocean warming

**Corresponding author name(s):** Dr Shuh-Ji Kao

**Editorial notes:**

**Transferred manuscripts**

This manuscript has been previously reviewed at another journal that is not operating a transparent peer review scheme. This document only contains reviewer comments and rebuttal letters for versions considered at *Nature Communications*.

**Reviewer comments & decisions:**

**Reviewer comments, first version:**

Reviewer #1 (Remarks to the Author):

Summary:
The authors have put together an interesting study investigating the interconnected effects of temperature and substrate concentration on ammonia oxidation rates across a marine transect from coastal to open ocean environments. They present convincing evidence highlighting the differences in optimal activity temperatures when substrate is either saturating or below saturating concentrations. In addition, they use inhibition experiments to show differential responses of AOA and AOB communities. With the finishing point being that coastal and open ocean ammonia oxidation rates will respond differentially to increasing ammonium concentrations and temperatures.

I think this manuscript is put together very well and presents a great data set that will push the field forward. Especially when thinking about the differential effects, warming will have on different niches in the large marine environment. However, I think that there are some key discussion points missing, and discussion points that can be flushed out more to increase the strength of this manuscript. Overall, I’m not sure the overall breakthrough presented in this study is novel enough for the readership of Nature Communications.

Main comments:

Line 95: I would argue that this generalization is not supported by Figure 1. Yes, the three sites with the highest Topt values (JLR1, 2, and 3) do have the highest ammonium concentrations (96, 24, and 5uM). However, the Topt of the other six sites does not follow this pattern. In place of this sentence maybe the authors could say something along the lines of “The highest Topt values were measured at sites with the highest ambient ammonium concentrations” or “ The sites with the highest ambient ammonium concentrations (>5uM), had Topt values well above the in situ temperatures measured at these sites. In contrast, all sites with lower ammonium concentrations (<550nM, except N3) had Topt values at or below the measured in situ temperatures.”

Lines 116-119: The authors bring up a great point here. Indeed, this may lead to an overestimation of the in situ Topt, simply because the lab experiments are generally carried out with saturating substrate concentrations. Could this be expanded upon to include a statement about how this may effect modeling of AOR in the ocean?

Line 120: I do not follow the authors train of thought here. Why does a higher sensitivity of the Km over the Vmax to temperature lead to a more predictable substrate regulated Topt? Than say if the reverse was true and the Vmax was more sensitive to temperatures changes. Unless, it is just the wording and the authors were trying to get at “With this data in hand, we may be able to model and predict substrate regulated Topt, and we do so below”? I believe the latter is the case.

Line 130: Any speculation here, as to why the DAMM model cannot predict AOR at temperatures above Topt-sat? Can anything be able to the model to account for this?

Line 177: This is another great point from the authors, but I feel it again needs clarification. What about the community composition explains the dome shaped and positive thermal responses? More specific details are needed here. Also I feel this is a great place to expand on the different thermal sensitivities of the AOB versus the AOA.

Line 193: What do the authors mean by the AOA at station N3 can adapt to the low ambient concentration of ammonium. How does this impact the observed dome shape? Do the authors mean that at this low ammonium concentration, the AOA are substrate saturated? If so, this is unclear. I believe this is what the rest of the paragraph gets at, but upfront it doesn’t make sense.

Line 233: I think the authors discussion would benefit from also mentioning that AOB have the potential to produce much higher yields of nitrous oxide than AOA. This is because they can perform “nitrifier denitrification” and enzymatically produce nitrous oxide from nitrite (where AOA can not, with a very recent exception Jung et al 2019). This may affect nitrous oxide emissions as increased nutrient concentrations select for more AOB in AO communities.

Not mentioned: Have the authors considered taking into account the specific affinity of their thermal kinetics, which take into account both the Km and the Vmax? This kinetic constant looks at the ability to scavenge substrate from dilute environments.

Small Changes:

Throughout: ammonia-oxidizing or ammonia oxidizing
Line 22: Increase to increased
Line 71: Light instead of Lights
Line 76: over three orders of magnitude not 4
Line 105: To further explore “the” substrate regulated…
Line 135: Q10Km isn’t consistently greater than Q10Vmax, it just is greater is this example
Line 142: for “the” onsite
Line 144: This sentence is confusing to pull the main points out of. It may have more impact being reworded and possibly split into two points.
Line 150: et al in italics
Line 164: “must” seems presumptive, maybe “could”, “probably”, or “very likely”?
Line 166: were presented to “are presented”
Line 167: comma after negative
Line 174: This kind of observation has been seen many times over both in the lab and with in situ communities. It should be supported with references and stated as reflecting what we known about the niche differentiation between the AOB and AOA.
Line 194: “~100%”
Line 188: and subsequent low Topt to “, resulting in a low Topt”.

Figure 3: I would suggest putting the uninhibited activity (grey bars) on the bottom of the graph so it’s easy to see the % AOR at each station. Also, when printed in black and white it is very hard to tell the difference between the bars and the white spaces in between the bars because all go to 100%. Maybe consider using either a dashed box for the inhibited amount, or a black and grey scale instead of white and grey.

Figure 4: The shaded area here needs to be described as the 0.4-4 degree increase in temperature mention in the IPCC study. In the x-axis it should be % of AOR “at” in situ…). Also in Line 599, it should be “is” denoted. Lastly, it may be worth mentioning in the discussion that with continued increasing temperatures (more than 10C) the reduction in offshore AO continues in what seems like a linear pattern, where the increase in AO seen in nearshore environments does not.

Supplementary Table 3: What are the units of the amoA copies?

Supplementary figure 2: Line 166 measurements “are” presented


Reviewer #2 (Remarks to the Author):

Overall I found this manuscript to be interesting and to provide exactly the type of multifactorial experimentation that needs to be conducted in order to understand and model multifactorial environmental change. However, there were multiple overarching deficiencies that absolutely must be addressed, as well as some specific minor issues.

Over-arching issues:

(1) Temperature intervals and context:
As the authors note in the manuscript, the temperature range and intervals used are large. Although there is substantial spatial temperature variation across the study sites, it is unclear how much temporal temperature variation occurs at a given location. It would be helpful to show average temperatures, and their seasonal variations, in the Supplement. (I assume what is shown in the Supp is just the temps at the time of experiments.) These temperature data will aid in comparing the values in experiments to actual conditions—i.e., what the AO communities experience, and have presumably adapted to.

This context is important when considering the implications of the findings for climate change. Given the relatively moderate change in temperature that occurs in the ocean due to the high specific heat capacity of water, the authors need to be very careful in how they interpret these data in the context of climate change. For example, Figure 4 shows substantial decreases in AOR with increasing temp, but the actual temp change via global warming is likely to be small compared to the width of the figure axis—i.e., a few degrees at most, vs. a 15 degree range here. So while the authors may observe a fundamental property of these AO communities, the implications for climate change need to be carefully crafted. One piece of information that would be useful is including any available information on how temperatures have changed and are projected to change in this region. If temperature change has been or expected to be large, this would make the data more relevant.

(2) Ammonia oxidation rate calculations:
The equation used for ammonia oxidation rate calculations is incorrect, as it does not account for the 15N enrichment of the ammonium pool. If this pool is always highly enriched, this won’t affect the rates strongly, but a slightly enriched NH4+ pool will substantially increase the calculated rates. See papers and book chapters by Bess Ward for the most commonly used formulation. This likely will not change the overall conclusions of the paper but may have a number of secondary implications.

(3) Lack of explanation for the fundamental mechanisms underlying the observations:
The paragraph starting on line 147 is very important for the manuscript, yet the beginning line states that: “The cause for substrate-independent Topt-sat remains unclear, therefore, further studies are needed to resolve the mechanisms behind this observation.” The text following is also not directly relevant—but may be peripherally relevant—because the authors are discussing the use of N (and P) as nutrients, not substrates, and the variable under investigation is a growth rate.

The authors need to provide a logical explanation (or several) for why Topt for AOR is affected by substrate concentrations. One thing to consider is that temperature and N do appear to co-vary somewhat across their sampling region.

(4) Community composition:
I understand the reasons for including the ATU treatments and I do think it adds some context to the paper. I would recommend adding the limited QPCR to Figure 3. However, where are the sequencing data? These are mentioned in the methods in the Supplement, but no data are provided. This may provide some useful information regarding particular groups or ecotypes that may be adapted to particular temperature or N concentration regimes.

(5) Overall conclusions:
Line 222: Please see my comments above about temperature. Are there any records of temperature change in the region that would be useful, in addition to using the global numbers?

Lines 223-230: This argument seems too simplistic to me. The main contribution of the paper is the combined investigation of N and temp change, not the independent effects. N is clearly increasing globally, esp in the W Pacific. Although I agree that temperature on its own could lead to decline offshore, temperature change is likely to be accompanied by a co-occuring change in N. The key then becomes the details. Is there a point that, with enough N, AOR start to increase with temperature change?

The authors could address this with their data and model. In fact, I would argue that Figure S2, or some similar figure, should be included in the paper. Why not generate a contour plot of some kind that shows the interactive influences of temp and N on the projected rates?

Lines 251-269: I think that this paragraph should probably start the final section. These caveats are very important in the extrapolating from the data, and this section also demonstrates that the authors are aware of the complimentary research approaches that are necessary to verify the findings reported here.

Smaller details:
Various reference numbers incorrect.

Lines 186-188: This statement seems to assume that the relative abundance of AOB is constant. If AOA become more prevalent, will this really hold?

Lines 311-313: Very confusing sentence, revise.

**Author rebuttal, first version:**

Reviewer #1

**Summary：**

The authors have put together an interesting study investigating the interconnected effects of temperature and substrate concentration on ammonia oxidation rates across a marine transect from coastal to open ocean environments. They present convincing evidence highlighting the differences in optimal activity temperatures when substrate is either saturating or below saturating concentrations. In addition, they use inhibition experiments to show differential responses of AOA and AOB communities. With the finishing point being that coastal and open ocean ammonia oxidation rates will respond differentially to increasing ammonium concentrations and temperatures.

I think this manuscript is put together very well and presents a great data set that will push the field forward. Especially when thinki**ng about the differential effects, warming will have on different niches in the large marine environment.** **However, I think that there are some key discussion points missing, and discussion points that can be flushed out more to increase the strength of this manuscript. Overall, I’m not sure the overall breakthrough presented in this study is novel enough for the readership of** Nature Communications.

**Reply:** Thanks for the reviewer’s constructive comments on this manuscript. In the new version, we conducted new thermal kinetics experiments for a nearshore AO community. These additional data allow us to quantify the thermal response in term of specific affinity, and to evaluate the warming effects on the competition advantages of nearshore and offshore AO communities. Therefore, we are now able to discuss additionally the evolutionary trends of niche space between AOAs and AOBs. **(lines 250 - 269)**

**Main comments:**

Line 95: I would argue that this generalization is not supported by Figure 1. Yes, the three sites with the highest T_opt_ values (JLR1, 2, and 3) do have the highest ammonium concentrations (96, 24, and 5uM). However, the T_opt_ of the other six sites does not follow this pattern. In place of this sentence maybe the authors could say something along the lines of “**The highest T_opt_ values were measured at sites with the highest ambient ammonium concentrations” or “The sites with the highest ambient ammonium concentrations (>5uM), had T_opt_ values well above the *in situ* temperatures measured at these sites. In contrast, all sites with lower ammonium concentrations (<550nM, except N3) had T_opt_ values at or below the measured *in situ* temperatures**.**”**

**Reply:** As suggestion, now we modified as “the highest T_opt_ values were observed at coastal sites with the highest ambient ammonium concentrations (Fig. 1)”. **(lines 95 - 96)**

Lines 116-119: The authors bring up a great point here. Indeed, this may lead to an overestimation of the *in situ* T_opt_, simply because the lab experiments are generally carried out with saturating substrate concentrations. **Could this be expanded upon to include a statement about how this may effect modeling of AOR in the ocean?**

**Reply:** This comment is very constructive. We added discussions about implications for AOR models. **(lines 120 - 126)**

“Some published biogeochemical models with a nitrification compartment have assumed that nitrification follows the Arrhenius relationship until temperature reaches the T_opt_^23,24^. However, the T_opt_ in these models is derived from pure laboratory cultures typically grown at saturating substrate concentrations (T_opt-sat_), which may cause an overestimation of rates in the warming ocean at ambient unsaturated substrate concentrations.”

Line 120: I do not follow the authors train of thought here. **Why does a higher sensitivity of the K_m_ over the V_max_ to temperature lead to a more predictable substrate regulated T_opt_? Than say if the reverse was true and the V_max_ was more sensitive to temperatures changes. Unless, it is just the wording and the authors were trying to get at “****With this data in hand, we may be able to model and predict substrate regulated T_opt_, and we do so below”? I believe the latter is the case.**

**Reply:** Thanks for the correction. Now we have modified relevant statements to: “To examine the interactive effects of temperature and substrate on AOR and T_opt_, we adapted the Dual Arrhenius and Michaelis-Menten kinetics model (DAMM) developed by Davidson *et al*.^25,26^. The temperature sensitivity of K_m_ (Q10_Km_) and V_max_ (Q10_Vmax_) (Fig. 2c - f) was applied.”; **(lines 127 - 130)** “For the T_opt_ model, T_opt_ increased as substrate concentration increases under the criterion of Q10_Km_ > Q10_Vmax_ (Supplementary Fig. 2). This criterion was fulfilled in both J1 and JLR4 cases (Fig. 2c - f).”. **(lines 138 - 140)**

We also explicitly stated that “some other microbes may respond differently to temperature, with Q10_Km_ ≤ Q10_Vmax_ for instance. This may lead to predictable yet unidirectional rate increases in response to warming (without substrate-regulated T_opt_) until the T_opt-sat_ is reached, regardless of substrate changes.”. **(lines 159 - 162)**

Line 130: Any speculation here, as to why the DAMM model cannot predict AOR at temperatures above T_opt-sat_? Can anything be able to the model to account for this?

**Reply:** We provide a potential mechanism to explain this in the text. That is “heat-impaired biological enzyme activity^27, 28^ might result in deviations from the relationship between V_max_ (K_m_) values and temperature from the Arrhenius law.” **(lines 146 - 147)**, and thus alter the predictive capacity of the DAMM model. Also, the model cannot be changed to account for the effect of thermal denaturation of enzymes.

Line 177: This is another great point from the authors, but I feel it again needs clarification. What about the community composition explains the dome shaped and positive thermal responses? More specific details are needed here. Also I feel this is a great place to expand on the **different thermal sensitivities of the AOB versus the AOA**.

**Reply:** We performed a set of high-resolution temperature manipulation experiments for AOB-dominated water (JLR4) sampled from an estuary. These AOBs living in a replete substrate environment were moved instantly after collection into a substrate-limited environment for a thermal kinetics test. These new data coupled with earlier results offer a comprehensive view on the thermal responses of the AO community, and significantly enhance the discussion regarding different AOR patterns (positive, negative, and dome-shape responses) as suggested. **(lines 209 - 225)**

“The low K_m_ for AOA indicates that the ambient substrate concentration can easily reach saturation state for AOA-dominated basin regions (see K_m_ values in Supplementary Table 2), thus, leading to a dome-shape thermal response pattern with the T_opt_ near to T_opt-sat_ likely around 26 °C (Fig. 1h, i; Fig. 3c), consistent with a report for an AOA isolate in lab culture^18^. Meanwhile, a T_opt-sat_ of ~ 30°C (Fig. 1a, b; Fig. 3d) falls within the range of 29 - 35°C reported for AOBs in pure culture^43,44^. Since the AOB-dominated JLR4 case showed a very wide T_opt_ as substrate varied, the negative response patterns (temperatures between 10 and 30°C) can be seen when the substrate was diluted to lower than the several uM level, mimicking the seaward advection of a water mass and subsequent substrate dilution. Accordingly, we speculate that the three types of AOR thermal response patterns observed in the field were a result of the combined thermal response patterns of AOB and AOA along the substrate gradient. Although this type of negative response pattern has never been reported before in the field, we observed it at shelf stations and in our dilution experiment. This suggests that when AOBs dominate the community AOR in the shelf transition zone (Fig. 4), community AOR may respond negatively to temperature rise due to a low T_opt_ under substrate stress.”

Line 193: What do the authors mean by the AOA at station N3 can adapt to the low ambient concentration of ammonium? How does this impact the observed dome shape? Do the authors mean that at this low ammonium concentration, the AOA are substrate saturated? If so, this is unclear. I believe this is what the rest of the paragraph gets at, but upfront it doesn’t make sense.

**Reply:** Now we state as following: “The low K_m_ for AOA indicates that the ambient substrate concentration can easily reach saturation state for AOA-dominated basin regions (see K_m_ values in Supplementary Table 2), thus, leading to a dome-shape thermal response pattern with the T_opt_ near to T_opt-sat_ likely around 26 °C (Fig. 1h, i; Fig. 3c), consistent with a report for an AOA isolate in lab culture^18^.”. **(lines 209 - 213)**

Line 233: **I think the authors discussion would benefit from also mentioning that AOB have the potential to produce much higher yields of nitrous oxide than AOA.** This is because they can perform “nitrifier denitrification” and enzymatically produce nitrous oxide from nitrite (where AOA can not, with a very recent exception Jung et al 2019). This may affect nitrous oxide emissions as increased nutrient concentrations select for more AOB in AO communities.

**Reply:** Thanks for this comment. We have now incorporated N_2_O emission into the discussion. **(lines 311 - 317)**

“In fact, field and laboratory culture studies showed that AOB have a greater potential than AOA to generate N_2_O. The N_2_O yield from AOB is approximately two times higher than that of AOA cultures^55^. Thus, in the nearshore where AOB dominated AOR, N_2_O production may be further enhanced. This would serve as a positive feedback for global warming, while the suppression of AOR in offshore regions creates a negative feedback.”

Not mentioned: Have the authors considered taking into account the specific affinity of their thermal kinetics, which take into account both the K_m_ and the V_max_? This kinetic constant looks at the ability to scavenge substrate from dilute environments.

**Reply:** Thank you for the constructive suggestion. The specific affinity value was provided in the Supplementary Table 2. We also now added discussions about this issue. **(lines 250 - 269)**

“Using the thermal responses of kinetic parameters, we try to evaluate warming effects on the competition between AOB and AOA. The specific affinity (α), the ratio of V_max_/K_m_, can be used to represent a microbe’s ability to scavenge substrate from dilute environments. Thus, microorganisms with higher α values are superior competitors when substrate is limiting. We found the specific affinity was higher for AOA-dominated J1 than for AOB-dominated JLR4 (Supplementary Table 2; Fig. 5a). Moreover, α values of AOB vary in a narrower range relative to those of AOA as temperature changes. The less variable α may reflect that AOB copes better with variable temperature in their habitat (Supplementary Table 2; Fig. 5a). In contrast, AOA are more sensitive to temperature change. As aforementioned in the DAMM model, the criterion to have substrate-dependent T_opt_ is Q10_Km_ > Q10_Vmax_, which also results in a reduction in α during warming for both AOA and AOB. Yet, the relative reduction in AOA specific affinity as temperature increases is more significant (Fig. 5a), suggesting AOAs are more competitive in low temperature environments relative to AOBs, and so may not be favored in a warming ocean. On the other hand, the specific affinity of AOBs is insensitive to temperature change, suggesting their adaptation to nearshore environments with greater temperature fluctuation. The seaward gradient in temperature fluctuations and ammonium concentrations determine the nitrifier community, thus, thermal response pattern of community AOR observed in the field.”

**Small Changes:**

Throughout: ammonia-oxidizing or ammonia oxidizing

**Reply:** All “ammonia-oxidizing”.

Line 22: Increase to increased

**Reply:** Modified.

Line 71: Light instead of Lights

**Reply:** Changed.

Line 76: over three orders of magnitude not 4

**Reply:** .Corrected.

Line 105: To further explore “the” substrate regulated…

**Reply:** Modified.

Line 135: Q10Km isn’t consistently greater than Q10Vmax, it just is greater is this example

**Reply:** We added “some other microbes may respond differently to temperature, with Q10_Km_ ≤ Q10_Vmax_ for instance. This may lead to predictable yet unidirectional rate increases in response to warming (without substrate-regulated T_opt_) until the T_opt-sat_ is reached, regardless of substrate changes.”. **(lines 156 - 160)**

Line 142: for “the” onsite

**Reply:** Changed.

Line 144: This sentence is confusing to pull the main points out of. It may have more impact being reworded and possibly split into two points.

**Reply:** Modified as “Overall, the DAMM model successfully predicts the entire thermal response curve, including rates and T_opt_, except when the manipulated temperatures exceed T_opt-sat_ (Fig. 3; Supplementary Fig. 2). AOR drops significantly when temperature is greater than the T_opt-sat_, so heat-impaired biological enzyme activity^27, 28^ might result in deviations from the relationship between V_max_ (K_m_) values and temperature from the Arrhenius law.”. **(lines 142 - 147)**

Line 150: et al in italics

**Reply:** Modified.

Line 164: “must” seems presumptive, maybe “could”, “probably”, or “very likely”?

**Reply:** Changed “must” to “very likely”.

Line 167: comma after negative

**Reply: N**ow we rephrase the sentence as “To further examine to what extent the community structure (proportions of AOA and AOB) might shape the thermal response patterns of community AOR observed in the field,”. **(lines 185 – 187)**

Line 174: This kind of observation has been seen many times over both in the lab and with *in situ* communities. It should be supported with references and stated as reflecting what we known about the niche differentiation between the AOB and AOA.

**Reply:** We have added references and statements about niche differentiation between the AOB and AOA. **(Lines 195 - 199)**

“A similar offshore pattern of community distribution was also observed in other regions, such as from the Pearl River estuary to the South China Sea^35^, and from the freshwater region of the Chesapeake Bay to the coastal and open ocean water column^36^. This pattern suggests that AOB strongly prefer substrate-replete niches, and vice versa for AOA^20,37^.”

Line 194: “~100%”

**Reply:** Changed “AOA contributed 100%” to “AOA-dominated”.

Line 188: and subsequent low Topt to “, resulting in a low Topt”.

**Reply:** The relevant sentence is now “…. community AOR may respond negatively to temperature rise due to a low T_opt_ under substrate stress.”. **(lines 224 - 225)**

Figure 3: I would suggest putting the uninhibited activity (grey bars) on the bottom of the graph so it’s easy to see the % AOR at each station. Also, when printed in black and white it is very hard to tell the difference between the bars and the white spaces in between the bars because all go to 100%. Maybe consider using either a dashed box for the inhibited amount, or a black and grey scale instead of white and grey.

**Reply:** Changed as reviewer’s suggestion (Figure 4 in new version).

Figure 4: The shaded area here needs to be described as the 0.4-4 degree increase in temperature mention in the IPCC study. In the x-axis it should be % of AOR “at” in situ…). Also in Line 599, it should be “is” denoted. **Lastly, it may be worth mentioning in the discussion that with continued increasing temperatures (more than 10C) the reduction in offshore AO continues in what seems like a linear pattern, where the increase in AO seen in nearshore environments does not.**

**Reply:** The figure and the caption have been modified as suggested (Figure 5 in new version). Indeed, the thermal response of AOR in nearshore beyond 10 °C increasement of temperature was not linearly as figured out by reviewer, therefore, we discussed the different AOR responses of <10 °C and > 10 °C separately. **(lines 275 - 281)**

“More specifically, the higher T_opt_ of these AO communities in nearshore regimes allows ocean warming to promote coastal AOR when the temperature change increment is < 10°C. The AOR then drops with further temperature increases, likely due to the impaired enzyme activity as discussed above. On the other hand, community AOR decreases linearly with warming (Fig. 5b) in offshore waters, mainly because current ambient temperatures are close to T_opt-sat_ for AOAs and/or higher than T_opt_ of AOB in low substrate state.”

Supplementary Table 3: What are the units of the *amo*A copies?

**Reply:** “copies/L” was introduced. The *amo*A copies data are presented in Figure 4 in new version and also provided in the dataset.

Supplementary figure 2: Line 166 measurements “are” presented

**Reply**: Corrected. (Figure 3 in new version)

Reviewer #2

**Summary：**

Overall I found this manuscript to be interesting and to provide exactly the type of multifactorial experimentation that needs to be conducted in order to understand and model multifactorial environmental change. **However, there were multiple overarching deficiencies that absolutely must be addressed,** as well as some specific minor issues.

**Reply**: Thanks for the helpful comments. In response, to this version we have 1) added discussion of ocean warming basing on multi-year observational temperature data in the study area; 2) converted ^15^N into ^14+15^N rates as suggested, and as predicted by the reviewer these temperature response patterns remain the same, not affecting associated conclusions we made previously; 3) provided potential mechanisms from the perspective of physiology for substrate-regulated T_opt_; 4) strengthened the discussion of the combined effect of substrate and temperature on community AOR.

**Over-arching issues:**

(1) Temperature intervals and context:

As the authors note in the manuscript, the temperature range and intervals used are large. Although there is substantial spatial temperature variation across the study sites, it is unclear how much temporal temperature variation occurs at a given location. **It would be helpful to show average temperatures, and their seasonal variations, in the Supplement.** (I assume what is shown in the Supp is just the temps at the time of experiments.) These temperature data will aid in comparing the values in experiments to actual conditions—i.e., what the AO communities experience, and have presumably adapted to.

This context is important when considering the implications of the findings for climate change. Given the relatively moderate change in temperature that occurs in the ocean due to the high specific heat capacity of water, **the authors need to be very careful in how they interpret these data in the context of climate change.** For example, Figure 4 shows substantial decreases in AOR with increasing temp, but the actual temp change via global warming is likely to be small compared to the width of the figure axis—i.e., a few degrees at most, vs. a 15 degree range here. So while the authors may observe a fundamental property of these AO communities**, the implications for climate change need to be carefully crafted**. One piece of information that would be useful is including any available information on how temperatures have changed and are projected to change in this region. If temperature change has been or expected to be large, this would make the data more relevant.

**Reply:** Thanks very much for the constructive comments.

We have put the seasonal temperature records into Supplementary Table 3, and added relevant discussions about ranges of temperature manipulations and future projections.

“The seasonal temperature (1m depth) ranges from 13 ~ 32 °C for the Jiulong River estuary^46^ (Supplementary Fig. 1; Supplementary Table 3). The average SST rise over the period of 1960 to 2010 in the studied area was 0.02 °C/y^47^, about half of the predicted maximum increase rate of the global mean. On the other hand, hydrography of the northern South China Sea is influenced by regional climate and local hydrodynamics, including the western boundary current intrusion, internal waves and monsoon wind^48^. The water temperature of the upper 200 m in the northern South China Sea ranges from 13 to 31 °C at different depths^48, 49^ and the mean temperature rise for the entire 200 m water column in the studied area was reported to be 0.09 °C/y from 1975 to 2005^50^. Generally speaking, the rise of water temperature in our study region is similar to or even larger than the global mean, suggesting this global warming rate can be applied in our study area.” **(lines 237 -249).**

“The manipulated temperature range set (~14 - ~34°C) was comparable with the natural seasonal temperature variation (13 – 32 °C in estuary and 13 - 31°C in sea basin) in marine AO microbial habitats (Supplementary Table 3). Meanwhile, at an increase rate of 0.02 °C (nearshore) or 0.09 °C (offshore) per year^11,12^, the temperature in 2100 will be ~2 °C (nearshore) and ~7 °C (offshore) higher than today, thus, the temperature range set by this study was also comparable to the temperature range of study regions expected in 2100 year (~15 - ~34°C in estuary and ~20 – ~38 °C in sea basin).” (**Supplementary Information lines 66 – 73)**

(2) Ammonia oxidation rate calculations:

**The equation used for ammonia oxidation rate calculations is incorrect**, as it does not account for the 15N enrichment of the ammonium pool. If this pool is always highly enriched, this won’t affect the rates strongly, but a slightly enriched NH4+ pool will substantially increase the calculated rates. See papers and book chapters by Bess Ward for the most commonly used formulation. This likely will not change the overall conclusions of the paper but may have a number of secondary implications.

**Reply:** The ammonia oxidation rates were recalculated following the equation below as suggested, and as predicted by the reviewer these temperature response patterns remain the same, not affecting statements we made previously.

$AOR= \frac{\Delta[^{15}NO_{X}^{-}]}{f_{NH_{4}^{+}}^{15}\times t}$,

where [^15^NO_X_^-^] is the change in concentration of ^15^NO_X_^-^ between the start and the end of the incubation due to ammonia oxidation, f ^15^NH_4_^+^ is the fraction of ^15^NH_4_^+^ labeled with ^15^N at the start of the incubation, and t is the length of incubation. (**lines 432 - 435)**

(3) Lack of explanation for the fundamental mechanisms underlying the observations:

The paragraph starting on line 147 is very important for the manuscript, yet the beginning line states that: “The cause for substrate-independent T_opt-sat_ remains unclear, therefore, further studies are needed to resolve the mechanisms behind this observation.” The text following is also not directly relevant—but may be peripherally relevant—because the authors are discussing the use of N (and P) as nutrients, not substrates, and the variable under investigation is a growth rate.

The authors need to provide a logical explanation (or several) for **why Topt for AOR is affected by substrate concentrations**. **One thing to consider is that temperature and N do appear to co-vary somewhat across their sampling region.**

**Reply:** This comment is well taken. We provided a possible explanation from the perspective of protein physiology. **(lines 148 – 158)**

“The substrate-dependent thermal optimum is attributable to the effect of temperature on biochemical kinetics and the structural stability of the enzymes. Increasing temperature promotes catalytic rate, thus, V_max_ increases due to increasing kinetic energy of reactants and rates of collision, as well as higher structural flexibility of enzymes^27,29^. However, higher structural flexibility (lower stability) also results in active sites with a reduced ability for ligand recognition and binding, therefore, lower kinetic efficiency. Accordingly, one important physiological response of an organism to rising temperature will be a reduction in substrate affinity (Supplementary Table 2; Fig. 5a), and thus higher substrate demands (i.e., higher K_m_ value) (Fig 2e, f)^25,29-31^. In other words, higher substrate levels help to compensate for enzyme structural stability losses and so promote growth rates at higher temperature.”

We also explicitly stated “Although these examples are functionally and taxonomically distant from AOA and ammonia-oxidizing bacteria (AOB), strong similarities in substrate/nutrient regulation characteristics may imply a similar mechanism of enzymatic thermal responses between chemoautotrophs and photoautotrophs.”. **(lines 172 - 176)**

According to the thermal tolerances and the competitive capabilities inferred from thermal responses of specific affinity of AOA and AOB, we also added implications on the environmental co-variance of temperature and substrate on niche differentiation and niche space changes in the warming ocean with anthropogenic N input.

“Yet, the relative reduction in AOA specific affinity as temperature increases is more significant (Fig. 5a), suggesting AOAs are more competitive in low temperature environments relative to AOBs, and so may not be favored in a warming ocean. On the other hand, the specific affinity of AOBs is insensitive to temperature change, suggesting their adaptation to nearshore environments with greater temperature fluctuation. The seaward gradient in temperature fluctuations and ammonium concentrations determine the nitrifier community, thus, thermal response pattern of community AOR observed in the field.” **(lines 261 – 269)**

“According to our observational data (Fig. 4), although AOB gene copies are much less, AOBs appear to be the major AOR contributor in the mid-to-lower estuary, coastal seas and even in shelf zones unless ammonium is down to the ~ hundreds of nM level. Thus, we speculate the niche space of AOBs may expand in the future in the land-ocean transition zone, because of their greater thermal adaptability and continuously increasing anthropogenic nitrogen inputs from continents.” **(lines 301 – 307)**

(4) Community composition:

I understand the reasons for including the ATU treatments and I do think it adds some context to the paper. I would recommend adding the limited QPCR to Figure 3. However, where are the **sequencing data**? These are mentioned in the methods in the Supplement, but no data are provided. **This may provide some useful information regarding particular groups or ecotypes that may be adapted to particular temperature or N concentration regimes.**

**Reply:** We apologize for the vague descriptions of our method in the previous supplementary materials, which misled the reviewer. In fact, we do not have sequencing data, we have only qPCR data. Now, we rephrase it (**See Supplementary Information: lines 14 - 42**) and put the qPCR data in the figure as suggested.

Based on the qPCR taxonomic data, we designed a new M-M experiment for AOB after diluting the substrate concentration, which illustrates the different thermal sensitivities of the AOBs versus the AOAs. These new data further confirm the importance of AO community in regulating the AOR thermal response pattern.

(5) Overall conclusions:

Line 222: Please see my comments above about temperature. Are there any records of temperature change in the region that would be useful, in addition to using the global numbers?

**Reply:** We have added the seasonal observational temperature data into the supplementary materials as suggested. Discussions regarding the temperature range experienced in the field and projected in future have been added. **(See above)**

Lines 223-230: This argument seems too simplistic to me. **The main contribution of the paper is** **the combined investigation of N and temp change, not the independent effects.** N is clearly increasing globally, esp in the W Pacific. Although I agree that temperature on its own could lead to decline offshore, temperature change is likely to be accompanied by a co-occuring change in N. The key then becomes the details. **Is there a point that, with enough N, AOR start to increase with temperature change?**

**Reply:** Yes, if N supply increases continuously to the open ocean, the offshore AOR in deeper depth might response positively before reaching the T_opt-sat_.

“Our results also suggest that the projected gradual increase in atmospheric deposition of anthropogenic nitrogen (including ammonium)^51-53^ may aid in the thermal adaption of ammonia-oxidizing microorganisms in ammonium-depleted offshore environments. However, most of the NH_4_^+^ from atmosphere will be utilized first by phytoplankton in the surface layer above the nitracline, due to a competitive advantage of phytoplankton toward NH_4_^+^ relative to nitrifers^17, 54^. If this ammonium was to be supplied directly to the niche of AOA, the offshore AOR might response positively to warming before reaching the T_opt-sat_. Note that AOAs prefer low temperature and cope with low substrate, so the peak AOR in open ocean generally appears near the nitracline^17^ where the vertical temperature gradient is also large. Therefore, the warming effect may also have a differential influence across the vertical scale.

In contrast, in the shallow nearshore dominated by AOBs additional inputs of anthropogenic NH_4_^+^ cannot further promote T_opt-sat_ to alter the empirical trend. Thus, a positive temperature response in nearshore regimes is more certain. According to our observational data (Fig. 4), although AOB gene copies are much less, AOBs appear to be the major AOR contributor in the mid-to-lower estuary, coastal seas and even in shelf zones unless ammonium is down to the ~ hundreds of nM level. Thus, we speculate the niche space of AOBs may expand in the future in the land-ocean transition zone, because of their greater thermal adaptability and continuously increasing anthropogenic nitrogen inputs from continents.” **(lines 287 – 307)**

The authors could address this with their data and model. In fact, I would argue that Figure S2, or some similar figure, should be included in the paper. Why not generate a contour plot of some kind that **shows the interactive influences of temp and N on the projected rates?**

**Reply**: We added the interactive influences of temperature and N on the projected rates into Fig. 3. Full scale AOR contour plots on variables of N(x) – T(y) for AOA and AOB were presented in Supplementary Fig. 2

Lines 251-269: I think that this paragraph should probably start the final section. These caveats are very important in the extrapolating from the data, and this section also demonstrates that the authors are aware of the complimentary research approaches that are necessary to verify the findings reported here.

**Reply:** We agree with the reviewer that it is very important to make these points, but think that because this paragraph focuses on future research directions, it is most appropriate to put this text at the end of the Discussion section.

**Smaller details:**

Various reference numbers incorrect.

**Reply:** Corrected.

Lines 186-188: This statement seems to assume that the relative abundance of AOB is constant. If AOA become more prevalent, will this really hold?

**Reply:** When AOBs dominate the community AOR in the shelf transition zone, the community AOR may respond negatively to temperature rise due to a low T_opt_ under substrate stress. If AOA become more prevalent, a dome-shaped pattern may be found (like J1 station).

Lines 311-313: Very confusing sentence, revise.

**Reply:** The ammonia oxidation rate calculation method was modified as suggested, thus the sentence “Ammonia oxidation rates presented here represent the production of ^15^NO_x_. We do not calculate the ^15^N production rates back to *in situ* rate in order to compare rates between low and high level of tracer addition in convenience.” was deleted.

**Reviewer comments, second version:**

Reviewer #1 (Remarks to the Author):

In my opinion the authors have done a great job addressing the comments of both reviewers. The manuscript changes along with the additional provided data, make this a stronger and more well-rounded manuscript. Below are just a few typesetting-like changes.

Line 91: “The Type III pattern was observed at station JLR3 (outer estuary), N2 (shelf), N3 and J1 (basin), for which the Topt of the AOR varied from 20°C to 29°C, with rates descending toward both higher and lower temperatures (Fig. 1c, g - i).” Rates cannot descend toward both high and lower temperatures. This should be reworded.
Line 100: This trend also holds true for Fig. 1d along with Fig. 1f,i.
Line 121: Switch “compartment” to “component”
Line 178: Maybe this sentence is missing “environment” or similar word after “off-shore”?
Line 257: “copes” to “cope”
Line 290: from “the” atmosphere
Line 293: “might response” to “may respond”


Reviewer #2 (Remarks to the Author):

I reviewed an earlier version of this manuscript and found this version to be much improved. In particular, I previously raised concerns about the context of the temperature manipulations, and the authors have very effectively dealt with my concerns. I also found the explanations for their findings to be more fully developed. Finally, individual issues -- such as rate calculations and molecular data -- have been been resolved.

Personally I find this to be a very interesting manuscript from multiple perspectives. First, the authors do an excellent job of laying out the broad significance of nitrification in the ocean, as well as the potential for biogeochemical feedbacks to both ocean warming and nitrogen enrichment. The combination of the sampling transect with the detailed experiments, the multifactorial global change perspective, and the in-depth analyses of the data are all well done. For all of these reasons, I have no additional comments.

**Author rebuttal, second version:**

Reviewer #1

In my opinion the authors have done a great job addressing the comments of both reviewers. The manuscript changes along with the additional provided data, make this a stronger and more well-rounded manuscript. Below are just a few typesetting-like changes.

**Reply:** Thanks for the reviewer’s appreciation of our work.

The typesetting-like errors have been corrected.

Line 91: “The Type III pattern was observed at station JLR3 (outer estuary), N2 (shelf), N3 and J1 (basin), for which the Topt of the AOR varied from 20°C to 29°C, with rates descending toward both higher and lower temperatures (Fig. 1c, g - i).” Rates cannot descend toward both high and lower temperatures. This should be reworded.

**Reply:** Changed to “….decreasing toward both higher and lower temperatures”

Line 100: This trend also holds true for Fig. 1d along with Fig. 1f, i.

**Reply:** We deliberately made the scales of the two y-axes in Fig.1d different to highlight the trend of *in situ* rate with temperature. In fact, the rate did not increase after substrate enrichment in Fig.1d.

Line 121: Switch “compartment” to “component”

**Reply:** Changed

Line 178: Maybe this sentence is missing “environment” or similar word after “off-shore”?

**Reply:** Added “environment”

Line 257: “copes” to “cope”

**Reply:** Changed.

Line 290: from “the” atmosphere

**Reply:** Added

Line 293: “might response” to “may respond”

**Reply:** Modified

Reviewer #2

I reviewed an earlier version of this manuscript and found this version to be much improved. In particular, I previously raised concerns about the context of the temperature manipulations, and the authors have very effectively dealt with my concerns. I also found the explanations for their findings to be more fully developed. Finally, individual issues -- such as rate calculations and molecular data -- have been been resolved.

Personally I find this to be a very interesting manuscript from multiple perspectives. First, the authors do an excellent job of laying out the broad significance of nitrification in the ocean, as well as the potential for biogeochemical feedbacks to both ocean warming and nitrogen enrichment. The combination of the sampling transect with the detailed experiments, the multifactorial global change perspective, and the in-depth analyses of the data are all well done. For all of these reasons, I have no additional comments.

**Reply**: Thanks for the reviewer’s appreciation of our work.

**Reviewer comments, third version:**

**Author rebuttal, third version:**
